# A hybrid method for assessing the perceived cultural value of cultural and creative products in e-commerce visual-textual presentation

**DOI:** 10.1038/s41598-026-58779-2

**Published:** 2026-06-23

**Authors:** Chen Chen, Xinyu Liu, Xian Zhou, Fangmin Cheng, Ping Liu, Yaxuan Zhou, Hang Zhao, Zhaojing Su

**Affiliations:** 1 School of Design, Xi’an Technological University, Xi’an, 710021 China; 2 Key Laboratory of Industrial Design and Ergonomics, Ministry of Industry and Information Technology, Northwestern Polytechnical University, Xi’an, 710072 China; 3 College of Arts, Shandong University of Science and Technology, Qingdao, 266590 China

**Keywords:** Cultural and creative products, perceived cultural value, e-commerce visual-textual presentation, multi-criteria decision analysis, socio-technical assessment, Cultural and media studies, Cultural and media studies, Information systems and information technology, Mathematics and computing

## Abstract

As the consumption of cultural and creative products (CCPs) increasingly shifts to e-commerce channels, consumers rely heavily on online visual-textual displays to perceive and compare cultural value, whereas systematic design-oriented methods for assessing such perceived value remain insufficient. To address this gap, this study proposes a hybrid method for assessing the perceived cultural value of CCPs in e-commerce visual-textual presentation. First, a three-domain indicator framework covering formal representation, usage inference, and meaning construction is developed by integrating hierarchical cultural semantics with narrative-structure organization, and refined through content validity testing. Second, a hybrid DEMATEL–CRITIC–MULTIMOORA model is used to integrate indicator influence and alternative-performance variation across indicators for weight determination, followed by multi-perspective alternative ranking. Using cultural aromatherapy burners as a case, Kendall’s concordance tests reveal evident divergence in expert judgments, with *W* = 0.128 and *p* = 0.797 for indicator interaction judgments, and *W* = 0.414 and *p* = 0.066 for indicator-level assessments of alternative performance. Within this context, the model outputs should be interpreted as structured perceived cultural value assessment results aggregated from heterogeneous expert judgments, rather than as objective measurements of products’ intrinsic cultural quality. External questionnaire validation demonstrates relatively high rank-order consistency between the model results and user-side assessments (Spearman’s *ρ* = 0.829; Kendall’s *τ* = 0.733). The proposed approach transforms implicit cultural semantic features of CCPs into observable and comparable evidence, providing a decision-support reference for competitor comparison, visual-textual presentation refinement, and CCP design practice.

**Supplementary Information:**

The online version contains supplementary material available at https://doi.org/10.1038/s41598-026-58779-2.

## Introduction

With the advancement of cultural digitalization and the improvement of online platform infrastructure, the consumption and dissemination of cultural and creative products (CCPs) are increasingly shifting from offline settings to platform-based channels such as e-commerce^1^. Unlike traditional museum souvenirs that are often purchased under immediate on-site stimulation, CCP selection in e-commerce environments relies more heavily on cross-product comparison based on primary images and visual-textual details^2^. Although short videos and livestreaming have become important forms of platform communication, they often introduce additional contextual cues, such as presenter narratives, interactive atmosphere, promotional discourse, and audiovisual effects, which may interfere with isolating the cultural meanings directly communicated by the product^3^. Visual-textual presentation is therefore more suitable for the standardized cross-product comparison examined in this study because it provides stable, revisitable, and product-centered evidence. Under such conditions, consumers usually judge from limited visual-textual information whether the cultural semantics embodied in a product can be clearly identified and understood, and whether the product is worth further comparison and purchase^4^. Therefore, what can be assessed in this context is not an objective or intrinsic cultural quality of the product itself, but the perceived cultural value communicated through e-commerce visual-textual presentation^5^. Such value needs to be operationalized into observable and comparable assessment evidence to inform product comparison and design decision-making.

Existing studies have accumulated knowledge on CCP assessment in terms of indicator development, evaluation methods, and empirical validation. Indicators have been constructed from dimensions such as aesthetics^6^, creativity^7^, functionality^8^, and cultural attributes^9^. From the perspective of consumer behavior, studies have also shown that design aesthetics, cultural identity, customer experience, and perceived value can influence purchase intention and product judgment^10,11^. These studies help explain how consumers respond to CCPs in platform environments, but many of them focus on broad psychological variables, market-related factors, or overall consumer attitudes, such as price, promotion, seller reputation, and purchase intention. As a result, their findings are not always directly convertible into design-oriented evaluation criteria. For design decision-making, the assessment of perceived cultural value under e-commerce visual-textual presentation requires indicators that have stable meanings, can be mapped onto observable visual-textual evidence, and can support comparison across product alternatives^12^. However, existing research has not yet provided a sufficiently operational framework for assessing perceived cultural value from the visual, functional, and symbolic cues available in e-commerce presentation.

This general limitation can be further specified in three aspects. First, existing assessment systems do not always decompose cultural semantics into clearly bounded and observable indicator domains. Some indicators may be conceptually meaningful, but their connotations remain broad or ambiguous, making them difficult to identify consistently from product images and textual descriptions. Second, existing evaluation models often rely on a single weighting or ranking logic and pay limited attention to inter-indicator relationships, heterogeneous expert preferences, and the integration of expert judgment with information derived from alternative performance. This may reduce the transparency and explanatory clarity of product comparison results. Third, many validation procedures depend on a single evidence source, such as expert assessment or questionnaire data alone, and rarely compare expert-structured model results with independent user-side evidence. Consequently, the external support for interpreting ranking results remains limited. These limitations indicate the need for a hybrid assessment method that can construct observable indicators, structure heterogeneous assessment information, and examine result interpretation through cross-source evidence.

To fill these gaps, this study develops an integrated analytical path covering indicator construction, hybrid assessment, and external examination. First, by integrating hierarchical cultural semantics with narrative-structure organization, a three-domain indicator framework is established, comprising formal representation, usage inference, and meaning construction. On this basis, secondary indicators are arranged according to consumers’ hierarchical cognitive logic, and observable evidence ranges are defined to facilitate perceived cultural value assessment in e-commerce visual-textual contexts. Second, a hybrid model integrating decision-making trial and evaluation laboratory (DEMATEL), criteria importance through intercriteria correlation (CRITIC), and multi-Objective Optimization on the basis of Ratio Analysis plus full multiplicative form (MULTIMOORA) is developed to combine expert-derived indicator influence with alternative-performance information for weight determination, and then compare product alternatives through multi-perspective ranking. Third, model outputs are compared with questionnaire-based user evidence to provide external support for interpreting the consistency of ranking results. Accordingly, this study makes three main contributions.

1. It reframes the cultural semantic expression of CCPs under e-commerce visual-textual presentation as an observable and comparable object for perceived cultural value assessment, and improves the structural coherence and interpretability of the indicator framework by combining hierarchical differentiation with cognition-oriented indicator organization.
2. It proposes a hybrid analytical path that clarifies the weighting and ranking process by integrating expert-derived indicator influence, alternative-performance information, and multiple ranking perspectives.
3. It introduces user-side evidence as an external reference for examining expert-derived model outputs, thereby supporting the interpretation of the findings and providing practical support for competitor comparison, visual-textual presentation refinement, and CCP design decision-making.

## Related works

### Shifting assessment requirements for CCPs in platform-based contexts

In platform-based contexts, the assessment of CCPs concerns how cultural value is organized, presented, and interpreted through digital interfaces. Take e-commerce platforms as an example, they are not neutral tools merely for displaying product content. Instead, page layout, image and text arrangement, user reviews, recommendation algorithms and traffic exposure together create a space for people to interpret and spread product value^13,14^. From a socio-technical systems perspective, consumers’ judgments of CCPs are not formed through a direct reading of inherent product attributes, but emerge from the interaction among technical interfaces, information cues, and subjective experience^15,16^. Service-dominant logic puts forward a clear core viewpoint. Product information posted on e-commerce pages represents a value proposition, rather than a static show of intrinsic product value^17^. Based on this understanding, consumers draw on various clues on the page to interpret, associate and jointly build the cultural meanings of products. Clearly, visuals and texts on web pages are more than passive information carriers. They serve as key media to generate, explain and compare cultural value across the platform.

For this reason, online visuals and texts lay a solid foundation for cultural value interpretation, as well as the comparative analysis of different CCPs. Limited by the way information is presented on these pages, we cannot assess the cultural value of such products with a single indicator. We need to combine visual styles, functional hints and symbolic meanings to reach a comprehensive conclusion. When people evaluate products through online content, ordinary goods are mainly judged by obvious traits such as function, material and price. CCPs work differently. People need to understand their cultural connotations from multiple angles, including appearance, graphic symbols, usage scenarios, symbolic implications and emotional resonance^9^. Once all these clues are organized and shown on e-commerce pages, cultural value can no longer be measured as a static product feature. It turns into a comprehensive perception, which users form based on what they can see, read and infer from the interface. Zeithaml’s means-end model suggests that perceived value is not a simple aggregation of product attributes, but an overall, multi-attribute evaluation formed through what consumers receive, interpret, and consider meaningful^18^. Throsby’s cultural economics framework further indicates that cultural value involves aesthetic, symbolic, spiritual, historical, and social dimensions that cannot be reduced to a single physical or economic attribute^19^. Accordingly, in e-commerce visual-textual presentation, the cultural value of CCPs is more appropriately conceptualized as perceived cultural value jointly shaped by visual, functional, and symbolic cues. Platform-based CCP evaluation should therefore shift from judging intrinsic cultural quality to assessing perceived cultural value and its observable cues.

### Frameworks for cultural-value interpretation and indicator organization

Regarding the formation and assessment of cultural value in CCPs, existing studies have approached this issue mainly from the perspectives of semiotics, emotional design, and hierarchical cultural semantics. Semiotics explains how cultural signs are encoded, recognized, communicated, and interpreted. It addresses issues such as sign recognizability, semiotic efficiency, and symbolic depth, thereby providing a basis for analyzing how cultural elements are translated into product graphics, patterns, colors, and visual symbols^20^. Emotional design emphasizes users’ experiential responses in visual perception, use comprehension, and reflective evaluation, and therefore supports the understanding of how the cultural value of CCPs is subjectively formed^21^. Hierarchical cultural semantics usually distinguishes cultural content into an outer material layer, a middle behavioral layer, and an inner spiritual layer, offering a layered perspective on how cultural resources are transformed into product forms, usage relationships, and deeper meanings^22–24^. In contrast to these product-level perspectives, macro-level cultural value frameworks, such as Hofstede’s cultural dimensions, primarily explain differences in cultural value orientations at national, organizational, or group levels and can serve as references for understanding cultural differences^25^. Taken together, these frameworks show that the assessment of cultural value in CCPs involves the expression and recognition of cultural signs, user experience, cultural layers, and value orientations. However, they differ in analytical level, evidence type, and operational applicability for product design evaluation.

These differences are particularly important for the present study, because perceived cultural value assessment under e-commerce visual-textual presentation requires a framework that can translate abstract cultural meanings into observable product-level assessment domains. Semiotic frameworks help explain the recognition and communication mechanisms of cultural signs, but when used alone as a primary structure, they cannot fully cover use-related inference and deeper value interpretation. Emotional design frameworks explain how cultural cues are received, experienced, and evaluated, yet they tend to focus on perceptual responses or experiential outcomes rather than a stable product-level indicator structure. Hofstede’s cultural dimensions are useful for explaining macro-level differences in cultural value orientations, but their analytical level does not directly correspond to the observable cultural semantics of specific CCPs presented through e-commerce images and text. By contrast, hierarchical cultural semantics links cultural content with different observable levels of product expression and is therefore more suitable as the basis for constructing the indicator framework in this study. Specifically, the outer material layer corresponds to visible formal expressions, such as form, color, material, texture, and graphic layout; the middle behavioral layer corresponds to functional organization, usage processes, contextual relevance, and the alignment between cultural meaning and contemporary use; the inner spiritual layer corresponds to thematic concepts, symbolic associations, emotional resonance, and value identification^26^. Based on this hierarchical relationship, this study organizes perceived cultural value assessment into three product-oriented assessment domains: formal representation, usage inference, and meaning construction. Formal representation emphasizes the visualization and recognizability of cultural cues; usage inference emphasizes the relationships between cultural meaning and product functions, contexts, and behavioral cues; and meaning construction emphasizes symbolic depth, emotional association, and value identification.

However, hierarchical cultural semantics primarily addresses what cultural content should be assessed; it does not fully explain how consumers encounter, connect, and interpret cultural cues in e-commerce visual-textual presentation. Existing product evaluation studies often construct indicators through element decomposition and form evaluative judgments using rating scales, weighting procedures, or multi-criteria aggregation methods^27,28^. Although this approach improves operational feasibility, it may treat indicators as static and mutually parallel items. In e-commerce visual-textual contexts, consumers usually follow information pathways formed by main images, titles, detail images, and descriptive texts, and gradually proceed through cue identification, cue integration, usage inference, and meaning judgment. Narrative structure provides a complementary explanatory mechanism for this process because meaning is not produced merely through the accumulation of isolated attributes, but is gradually generated through contextual unfolding, action inference, and outcome interpretation^29,30^. Relevant studies further indicate that narrative structures can support design process organization, product meaning construction, and coherent user understanding^31,32^. Accordingly, this study introduces narrative organization on the basis of hierarchical cultural semantics to coordinate the cognitive sequence and evidential relationships among secondary indicators within each assessment domain. This enables perceived cultural value assessment to have not only a clear content hierarchy, but also an organizational structure that better matches the visual-textual reading logic of e-commerce environments.

### MADM methods for CCP assessment and alternative comparison

Multi-criteria decision analysis (MCDA) provides a broad methodological framework for addressing decision problems involving multiple indicators, objectives, or preferences^33^. From a methodological perspective, MCDA does not aim to calculate absolute or objectively verifiable “true values”. Rather, it provides structured procedures for clarifying decision problems, documenting judgment processes, comparing alternative performance, and managing trade-offs among criteria. Belton and Stewart’s MCDA framework emphasizes that multi-criteria methods help decision-makers organize trade-offs and make judgments explicit when multiple, and often conflicting, criteria are involved^33^. This research approach fits well with the assessment of CCPs. Decision-makers differ greatly in cultural literacy, aesthetic taste, practical demands and the ability to grasp symbolic meanings. For this reason, we cannot assess such products against one single objective standard. Instead, the work is a structured comparative study. It involves multiple indicators, diverse personal preferences and a range of assessed objects. As an important branch of MCDA, multi-attribute decision making (MADM) focuses on evaluating and ranking limited alternatives across diverse attributes. At present, it is widely used in the evaluation and selection of CCPs.

Among these, methods such as the analytic hierarchy process (AHP), best-worst method (BWM), DEMATEL, and their variants are frequently used to determine indicator importance. Ranking methods such as the technique for order preference by similarity to an ideal solution (TOPSIS), vlseKriterijumska optimizacija I kompromisno resenje (VIKOR), tomada de decisão interativa e multicritério (TODIM), grey system approaches, and fuzzy methods are commonly used for alternative comparison and uncertainty handling. Based on these, prior studies have proposed evaluation models such as AHP–TOPSIS^34^, Kano–AHP–TOPSIS^35^, Kano–AHP–DEMATEL–VIKOR^36^, intuitionistic fuzzy BWM–TODIM with group consensus^27^, and AHP–grey system theory–fuzzy evaluation^37^ for CCP or broader product evaluation contexts. These studies demonstrate the applicability of MADM to product evaluation. However, perceived cultural value assessment of CCPs under e-commerce visual-textual presentation poses more specific methodological requirements. Indicators may be interrelated, expert judgments may be heterogeneous, and the determination of indicator importance needs to consider both expert judgments on inter-indicator influence and the differentiated performance of alternatives across indicators. Alternative ranking should also avoid excessive dependence on a single aggregation logic. In addition, the present task differs from personalized prediction models based on large-scale user behavioral data. Such models mainly serve preference prediction and content matching, whereas this study focuses on an interpretable, indicator-based comparison of a small set of CCP alternatives under controlled visual-textual evidence. Therefore, a structured assessment method is needed to make indicator relationships, weighting sources, and ranking logic explicit.

In response to these methodological requirements, this study develops a DEMATEL–CRITIC–MULTIMOORA hybrid assessment model within an MADM problem framework to support structured perceived cultural value assessment of CCPs. Specifically, DEMATEL is used to characterize complex interrelationships among assessment indicators and to extract indicator influence structures from expert judgments, thereby supporting the interpretation of relationships among formal representation, usage inference, and meaning construction. CRITIC is used to extract information on the variation and contrast of alternative performance across indicators, thereby supplementing the information that may be insufficient when relying only on expert-derived inter-indicator influence judgments^38^. On this basis, MULTIMOORA compares alternatives from multiple aggregation perspectives through the ratio system, reference point approach, and full multiplicative form, helping reduce the dependence of ranking results on a single assessment logic^39^. Thus, these methods are not integrated to generate an objective score for “cultural quality”; rather, they form a hybrid assessment model that supports inter-indicator relationship analysis, use of alternative-performance information, and multi-perspective alternative comparison. Given that the model outputs are primarily derived from structured expert judgments, their interpretation also requires external reference to user-side evidence.

### Research gap

In summary, existing studies have provided theoretical and methodological foundations for CCP perceived cultural value assessment from different perspectives, including platform-based consumer judgment, cultural value perception, cultural semantic expression, and multi-attribute assessment methods. However, these research streams have not yet been sufficiently integrated into a design-oriented assessment framework for perceived cultural value under e-commerce visual-textual presentation. Specifically, existing studies still lack a systematic pathway for constructing indicators that connect hierarchical cultural semantics, observable visual-textual evidence, and consumers’ cognitive organization processes. In addition, few studies have simultaneously addressed inter-indicator relationships, heterogeneous expert judgments, alternative-performance information, and multi-perspective comparison within a unified assessment framework. Existing research also rarely compares structured expert-derived results with independent user-side evidence, leaving model outputs with limited external reference for interpretation.

To address these gaps, this study responds from three aspects: indicator construction, hybrid assessment, and result interpretation. First, a three-domain indicator framework is established by integrating hierarchical cultural semantics with narrative-structure organization. Second, inter-indicator relationships, expert judgments, and alternative-performance information are incorporated into a DEMATEL–CRITIC–MULTIMOORA hybrid assessment model for multi-perspective alternative comparison. Third, user survey evidence is introduced during the result interpretation stage as an external reference. Through this analytical pathway, cultural semantic cues embedded in e-commerce visual-textual presentation can be transformed into interpretable and comparable assessment results, thereby providing more traceable decision support for CCP alternative comparison and visual-textual presentation refinement.

## Development of an indicator framework for perceived cultural value assessment

This section clarifies what is assessed in perceived cultural value assessment by translating the abstract construct of perceived cultural value into observable primary domains, secondary indicators, and evidence domains applicable to e-commerce visual-textual materials.

### Construction of primary assessment domains based on hierarchical cultural semantics

To systematically evaluate the perceived cultural value of CCPs under e-commerce visual-textual presentation, this study builds on the three-level cultural structure commonly used in culturally oriented product design research and further translates this hierarchy into product-oriented assessment domains from a design-observability perspective. In this study, perceived cultural value is not treated as an objective or intrinsic cultural quality, but as the extent to which cultural meanings can be observed, inferred, and interpreted through product images and textual descriptions. Accordingly, the assessment domains are organized into three primary indicators: formal representation (*C*^1^), usage inference (*C*^2^), and meaning construction (*C*^3^). These three indicators describe different analytical layers of perceived cultural value in platform-based visual-textual contexts. Specifically, formal representation (*C*^1^) corresponds to the outer and directly observable layer of cultural semantic expression. It captures whether cultural cues are embodied in the product’s explicit design attributes and can be directly identified from visual-textual information. Usage inference (*C*^2^) corresponds to the intermediate layer in which cultural expression is connected with product use. It captures whether consumers can infer product purpose, mode of use, and the fit between cultural elements and the product carrier on the basis of such cues. Meaning construction (*C*^3^) corresponds to the inner and interpretive layer of perceived cultural value. It captures the deeper cultural understanding and identification that may be supported by the preceding two dimensions, including the interpretation of cultural connotations, value propositions, and identity-related meanings.

Because these three dimensions may overlap in actual assessment, their measurement domains were further separated to reduce ambiguity. In this study, *C*^1^ is restricted to directly observable design cues, *C*^2^ to consumers’ inferences about use-related and carrier-fit relationships, and *C*^3^ to deeper cultural understanding and identification outcomes. This boundary setting allows the indicator framework to remain focused on presentation-based perceived cultural value assessment, rather than expanding into a broad assessment of cultural authenticity, historical accuracy, or overall product quality.

### Construction of secondary indicators based on narrative-structure organization

Building on the primary indicators, this study developed secondary indicators to specify the assessment content within each dimension. Rather than treating perceived cultural value assessment as a static decomposition of outcomes, candidate items were identified through literature analysis (LA) and a public review corpus from e-commerce platforms (PRC), denoted as $\:{C}^{l}=\left\{{C}_{1}^{l},{C}_{2}^{l},\dots\:,{C}_{r}^{l},\dots\:{C}_{R}^{l}\right\}$, where $\:{C}_{r}^{l}$ represents the *r*-th secondary indicator under the *l*-th primary indicator. Candidate generation considered prior assessment dimensions, the cues available under e-commerce visual-textual presentation and the range of content that non-expert respondents could reasonably judge. Accordingly, candidate indicators were required to be expressible through observable visual-textual cues rather than abstract cultural terminology alone.

Because consumers’ evaluation of CCPs in e-commerce contexts is usually supported by product images, textual descriptions, usage cues, and cultural symbols, this study introduced narrative-structure organization not to propose a fixed or irreversible psychological sequence of consumer cognition, but to provide an analytical framework for organizing the observable evidence domains of perceived cultural value assessment indicators. Specifically, narrative structure helps explain how cultural cues can be connected from formal presentation, to use-related association, and further to symbolic and cultural interpretation. This organization reduces the ambiguity of indicator boundaries and improves the structural coherence of the secondary indicator system. Accordingly, the indicator-generation process was organized around four analytical nodes: information positioning, cue integration, reasonable inference, and overall judgment, denoted as $\:S=\left\{{S}_{1},{S}_{2},{S}_{3},{S}_{4}\right\}$. These nodes served as constraints for secondary indicator generation and organization, rather than as assumptions about a universal consumer cognition pathway. Based on this framework, candidate items were screened according to four rules.

1. Exclusive affiliation: each secondary indicator belongs to only one primary indicator;
2. Exclusive stage assignment: each secondary indicator corresponds to only one narrative stage;
3. Evaluability: each secondary indicator must be clearly defined and judgeable by non-expert respondents based on platform visual-textual information;
4. Evidence-domain constraint: each candidate indicator is assigned a primary evidence source to reduce informational redundancy.

Following these rules, the research team screened, classified, and standardized the initial secondary items, resulting in 15 candidate secondary indicators under the three primary indicators. Table 1 presents their detailed information. Although these items broadly covered the main content of perceived cultural value assessment, further refinement was still needed in terms of conceptual boundaries and evidence domains. Therefore, the 15 candidate indicators were not directly included in the formal assessment, but were further screened and revised through expert content validity testing to determine the final secondary indicator system.

**Table 1:** Candidate secondary indicators and their measurement information.

Primary indicator	Secondary indicator	Narrative stage	Indicator definition	Evidence domain	Source
Formal representation (*C*^1^)	Morphological composition ($\:{C}_{1}^{1}$)	*S*_1_	Stable form structure supporting recognition of cultural prototypes.	Massing proportion, contour framework, compositional order	LA^5^ and PRC
	Color scheme ($\:{C}_{2}^{1}$)	*S*_2_	Coordinated color hierarchy supporting functional zoning and cultural consistency.	Dominant color, area proportion, color contrast	LA^40^ and PRC
	Material expression ($\:{C}_{3}^{1}$)	*S*_3_	Material and surface cues supporting quality expectation and cultural texture.	Material category, surface treatment, texture and gloss	LA^5^ and PRC
	Graphic symbols ($\:{C}_{4}^{1}$)	*S*_4_	Coherent graphic symbols fitting product type and overall style.	Patterns, typeface, symbols	LA^41^ and PRC
	Craft details ($\:{C}_{5}^{1}$)	*S*_3_	Reasonable detail treatment improving completeness and avoiding roughness.	Component joints, edge transitions, craftsmanship traces	LA^5^ and PRC
Usage inference (*C*^2^)	Functional logic ($\:{C}_{1}^{2}$)	*S*_1_	Coherent functional structure linking key components and supporting use flow.	Functional zones, key components, operation path	LA^22,41^
	Context fit ($\:{C}_{2}^{2}$)	*S*_2_	Feasible usage context consistent with product features and spatial constraints.	Placement form, maintainability, spatial boundaries	LA^7^ and PRC
	Interaction path ($\:{C}_{3}^{2}$)	*S*_3_	Recognizable interaction path supporting expected feedback and basic fault tolerance.	Entry controls, operational feedback, tolerance cues	PRC
	Cultural carrier fit ($\:{C}_{4}^{2}$)	*S*_4_	Reasonable match between cultural carrier and product category, use, and context.	Semantic elements, product usage, mapping rationale	LA^9^
	Experience expectation ($\:{C}_{5}^{2}$)	*S*_4_	Consistent experiential cues shaping usage expectations and product positioning.	Appearance cues, visual-textual information, product positioning	LA^41,42^ and PRC
Meaning construction (*C*^3^)	Cultural symbols ($\:{C}_{1}^{3}$)	*S*_1_	Identifiable symbols supporting theme recognition and initial interpretation.	Symbol entry points, referential links, interpretive cues	LA^43^ and PRC
	Value narrative ($\:{C}_{2}^{3}$)	*S*_1_	Coherent narrative structure organizing value claims and maintaining internal consistency.	Core framework, supporting cues, structural consistency	LA ^42,44^
	Emotional resonance ($\:{C}_{3}^{3}$)	*S*_2_	Perceptible emotional cues supporting resonance and narrative alignment.	Emotional triggers, atmosphere building, resonance expression	LA^45^ and PRC
	Identity recognition ($\:{C}_{4}^{3}$)	*S*_3_	Clear identity cues supporting symbolic expression of identity and values.	Identity markers, value stance, contextual expression	LA^11,46^
	Contemporary translation ($\:{C}_{5}^{3}$)	*S*_4_	Smooth contemporary translation preserving core cultural semantics and fitting current aesthetics.	Translation strategies, contemporary aesthetics, semantic retention	LA^37^

*S*_1_–*S*_4_ denote the four narrative stages, namely information positioning, cue integration, reasonable inference, and overall judgment. LA = literature analysis; PRC = public review corpus from e-commerce platforms.

### Content validity testing

To improve the content appropriateness and structural rigor of the candidate indicator system, this study employed the content validity index (CVI) for expert quantitative assessment to screen and revise the 15 candidate secondary indicators. Following the COSMIN framework, content validity was examined in terms of relevance (*CVI*_Rel_), clarity (*CVI*_Cla_), and representativeness (*CVI*_Rep_)^47^. In addition, because this study focuses on online assessment under e-commerce visual-textual presentation, operability (*CVI*_Ope_) was introduced to assess whether an indicator could be rated consistently under the given information constraints.

Specifically, *CVI*_Rel_ reflects whether an indicator directly corresponds to the core meaning of its primary indicator; *CVI*_Cla_ reflects whether the indicator is clearly defined and understandable without professional background; *CVI*_Rep_ reflects whether it covers a necessary and relatively independent sub-dimension while avoiding substantial overlap with other items; and *CVI*_Ope_ reflects whether evaluators can rate it stably based only on e-commerce visual-textual information. Experts rated each indicator on a four-point scale (1 = not appropriate, 2 = somewhat inappropriate, 3 = somewhat appropriate, 4 = highly appropriate). Scores of 3 and 4 were treated as acceptance, whereas 1 and 2 were treated as non-acceptance. Following the literature^48^, this study takes *CVI*_Rel_ as an example and reports item-level CVI (I-CVI) calculation in Eq. (1). To reduce the effect of chance agreement on the I-CVI, a modified coefficient κ^*^ was further introduced, thereby providing a more conservative quantitative basis for the screening and revision of secondary indicators (see Eqs. (2)-(3)).

1 $$\:I-CV{I}_{\mathrm{R}\mathrm{e}\mathrm{l}}=\frac{{N}_{\mathrm{R}\mathrm{e}\mathrm{l}}}{N}$$

where *N*_Rel_ represents the number of experts who rated the indicator as 3 or 4 on *CVI*_Rel_, and *N* is the total number of experts.

2 $$\:{\kappa\:}^{\ast\:}=\frac{I-CV{I}_{\mathrm{R}\mathrm{e}\mathrm{l}}-{P}_{c}}{1-{P}_{c}}$$

where *P*_*c*_ is the probability of chance agreement, which can be calculated using Eq. (3).

3 $$\:{P}_{c}=\frac{N!}{{N}_{\mathrm{R}\mathrm{e}\mathrm{l}}!\left(N-{N}_{\mathrm{R}\mathrm{e}\mathrm{l}}\right)!}\times\:5N$$

To examine the content validity of the preliminary indicator framework for perceived cultural value assessment, this study invited eight experts from the fields of cultural studies, design, marketing research, and user research to form the review panel. All experts had more than 10 years of professional experience in either designing CCPs or conducting in-depth consumer research on such products, enabling them to make informed judgments on indicator meaning and contextual applicability. The review focused on the 15 candidate secondary indicators, and the I-CVI together with the modified agreement coefficient was calculated for each item based on Eqs. (1)–(3). The same decision rules were applied in both rounds:

1. Delete: an indicator was deleted if its relevance or representativeness was recognized by no more than five experts;
2. Retain: an indicator was retained if both relevance and representativeness were recognized by at least seven experts;
3. Revise: all other cases, including boundary items and those with insufficient clarity or operability, were classified for revision.

In the first round, $\:{C}_{5}^{1}$ (craftsmanship details), $\:{C}_{5}^{2}$ (experience expectations), and $\:{C}_{1}^{3}$ (cultural symbols) were removed because they were endorsed by no more than five experts in relevance or representativeness. Most of the remaining indicators were either borderline or showed deficiencies in clarity or operability and were therefore revised. Accordingly, the research team refined the indicator definitions, evidence domain prompts, and statement boundaries, and recoded the retained indicators accordingly. In the second round, the revised indicators were reassessed using the same rules, and consistency in relevance and representativeness was further improved. Appendix A1 reports the first-round content validity results for the candidate indicator system presented in Table 1, including κ^*^, decisions, and revision suggestions. Appendix A2 reports the second-round results for the revised indicator set, including the refined indicator names, definitions, evidence domains, κ^*^, and final decisions. Table 2 presents the final secondary indicator system for perceived cultural value assessment established after the second-round review and subsequent refinement by the research team. The evidence domains specify the observable boundaries of each indicator and offer concrete visual-textual references for interpretation. They are not intended to verify the historical authenticity or factual accuracy of cultural sources. Instead, they indicate what types of product images, textual descriptions, and presentation cues can support judgments about whether cultural expression is clear, consistent, and interpretable. For example, the evidence domain of “contemporary translation” directs attention to whether traditional cultural elements are perceptibly transformed through contemporary forms, materials, functions, or usage scenarios, rather than to whether the cultural source itself is historically authentic.

**Table 2:** Assessment indicators and evidence domains for perceived cultural value.

Primary indicator	Secondary indicator	Narrative stage	Indicator definition	Evidence domain
Formal representation (*C*^1^)	Morphological composition ($\:{C}_{1}^{1}$)	*S*_1_	Stable form structure supporting recognition of cultural prototypes.	Massing proportion (length–width ratio, thickness); contour framework (outer contour, axial relations); compositional order (hierarchy, inner–outer relation)
	Color organization ($\:{C}_{2}^{1}$)	*S*_2_	Coordinated color hierarchy supporting primary–secondary differentiation and stylistic consistency.	Primary–secondary color scheme; color-block proportion; color contrast (warm–cool contrast; light–dark contrast)
	Material expression ($\:{C}_{3}^{1}$)	*S*_3_	Material and process cues supporting texture expectation and cultural character.	Material and process cues (material type); texture cues (texture type, texture thickness); gloss cues (glossy surface, matte surface)
	Graphic composition ($\:{C}_{4}^{1}$)	*S*_4_	Ordered graphic symbols fitting layout rules and overall style.	Graphic motifs (geometric patterns, animal patterns); layout mode (centered layout, symmetry); organizational rules (repetition, alignment)
Usage inference (*C*^2^)	Functional coherence ($\:{C}_{1}^{2}$)	*S*_1_	Coherent functional structure linking key components and assemble relations.	Functional zones (carrying area, support area); key components (operation parts, connecting parts); connection modes (buckle, insertion)
	Use guidance ($\:{C}_{2}^{2}$)	*S*_2_	Controllable use process with explicit feedback and basic tolerance.	Entry controls (button, handle); operational feedback (prompt marks, scale position); tolerance cues (guiding structure, limiting structure)
	Environmental fit ($\:{C}_{3}^{2}$)	*S*_3_	Feasible fit with usage context, maintenance and safety boundaries.	Placement form (contact area, occupied space); maintenance mode (disassembly, replacement); safety boundaries (rounded corners, shielding)
	Cultural carrier fit ($\:{C}_{4}^{2}$)	*S*_4_	Appropriate fit between cultural expression and product carrier.	Cultural cues (object prototype, cultural source); carrier use (display, gift); mapping expressions (implied meaning, symbolism)
Meaning construction (*C*^3^)	Value narrative ($\:{C}_{1}^{3}$)	*S*_1_	Coherent symbolic and value cues across touchpoints.	Symbolic cues (inscription, symbolic pattern); value claims (auspiciousness, inheritance); touchpoint consistency (alignment of name, copy, and image)
	Emotional resonance ($\:{C}_{2}^{3}$)	*S*_2_	Emotional cues supporting atmosphere and resonance.	Emotional cues (emotion words, evaluative words); atmosphere building (scene, lighting); emotional articulation (healing, companionship)
	Identity recognition ($\:{C}_{3}^{3}$)	*S*_3_	Identity cues reinforcing value stance and contextual expression.	Identity markers (regional name, craft name); stance expression (combination of tradition and modernity); contextual expression (office, home, social settings)
	Contemporary translation ($\:{C}_{4}^{3}$)	*S*_4_	Translation strategy preserving core semantics while fitting contemporary aesthetics.	Translation articulation (extraction, reconstruction); semantic retention (core imagery, cultural symbolism); contemporary vocabulary (modern materials, modern interaction)

## Materials and methods

### Overview of the methodological framework

**Fig. 1:**
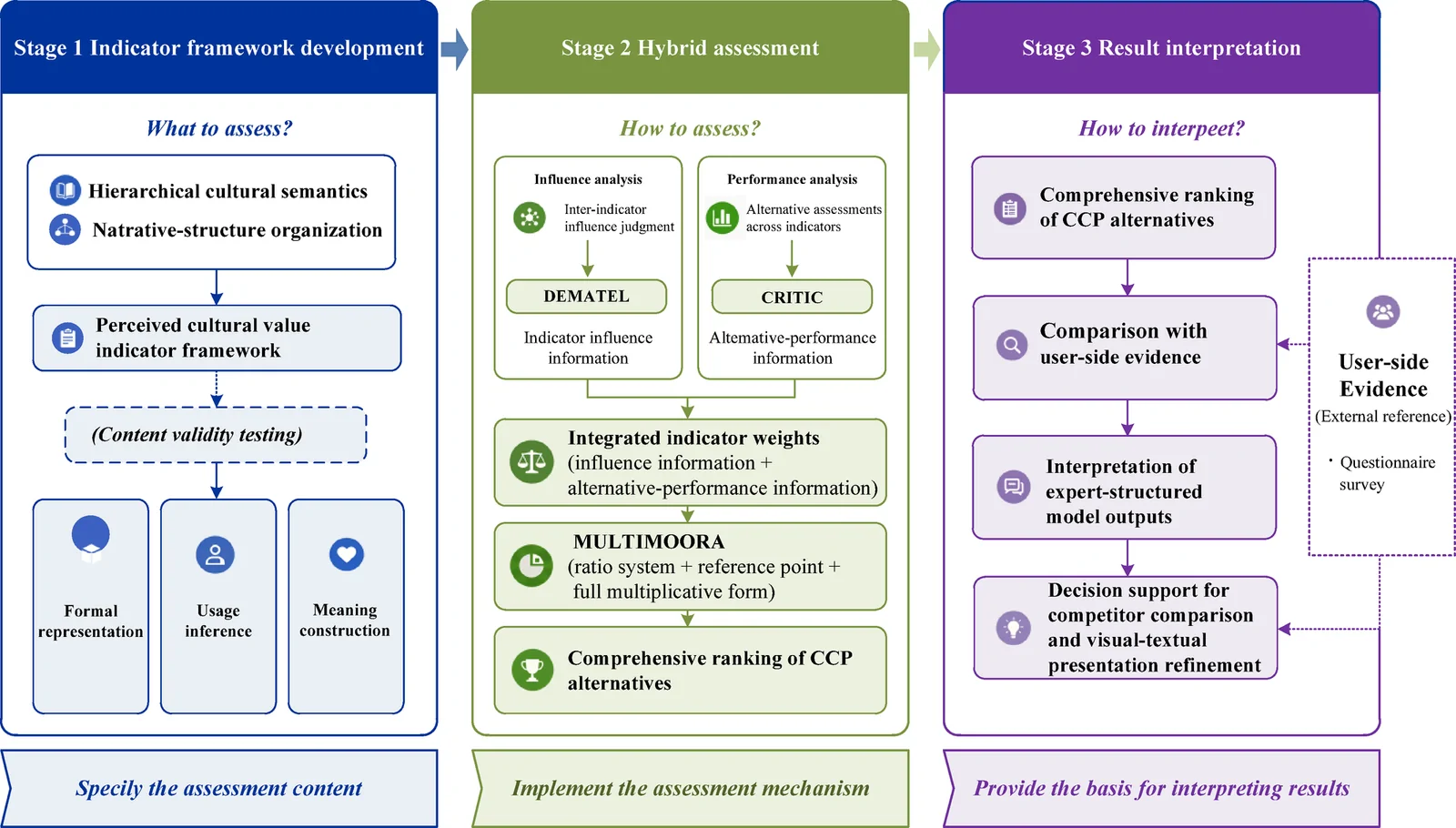
Overall methodological framework for perceived cultural value assessment. Solid arrows indicate the main analytical flow across the three stages. Dashed elements denote supporting procedures or external reference evidence, including content-validity refinement during indicator development and user-side evidence for result interpretation.

Perceived cultural value assessment of CCPs under e-commerce visual-textual presentation requires clarification of three interrelated issues: assessment content, assessment mechanism, and the basis for result interpretation. First, perceived cultural value needs to be translated into observable assessment content. Building on hierarchical cultural semantics and narrative-structure organization, we define three product-oriented domains: formal representation, usage inference, and meaning construction, which form the core of the indicator framework. Second, within the established indicator framework, the assessment mechanism addresses three key requirements: accounting for inter-indicator influence, capturing differentiated alternative performance across indicators, and enabling multi-perspective alternative comparison. These are implemented via the DEMATEL–CRITIC–MULTIMOORA hybrid model. Third, since model outputs are primarily derived from structured expert judgments, the ranking results require user-side evidence (e.g., questionnaire survey data) as an external reference to support interpretation and validation. Based on this logic, the overall methodological framework of this study is constructed as shown in Fig. 1.

Figure 1 summarizes the analytical pathway from indicator framework development to hybrid assessment and result interpretation. The indicator framework development stage defines the assessment content of perceived cultural value, supported by content validity testing. The hybrid assessment stage adopts the proposed combined model to synthesize multi-source information and produce a full ranking of CCP alternatives. The result interpretation stage introduces user-side evidence as an external reference, enabling comparison with expert-derived results and providing decision support for competitor analysis and visual-textual presentation refinement.

### Implementation of the hybrid assessment model

**Fig. 2:**
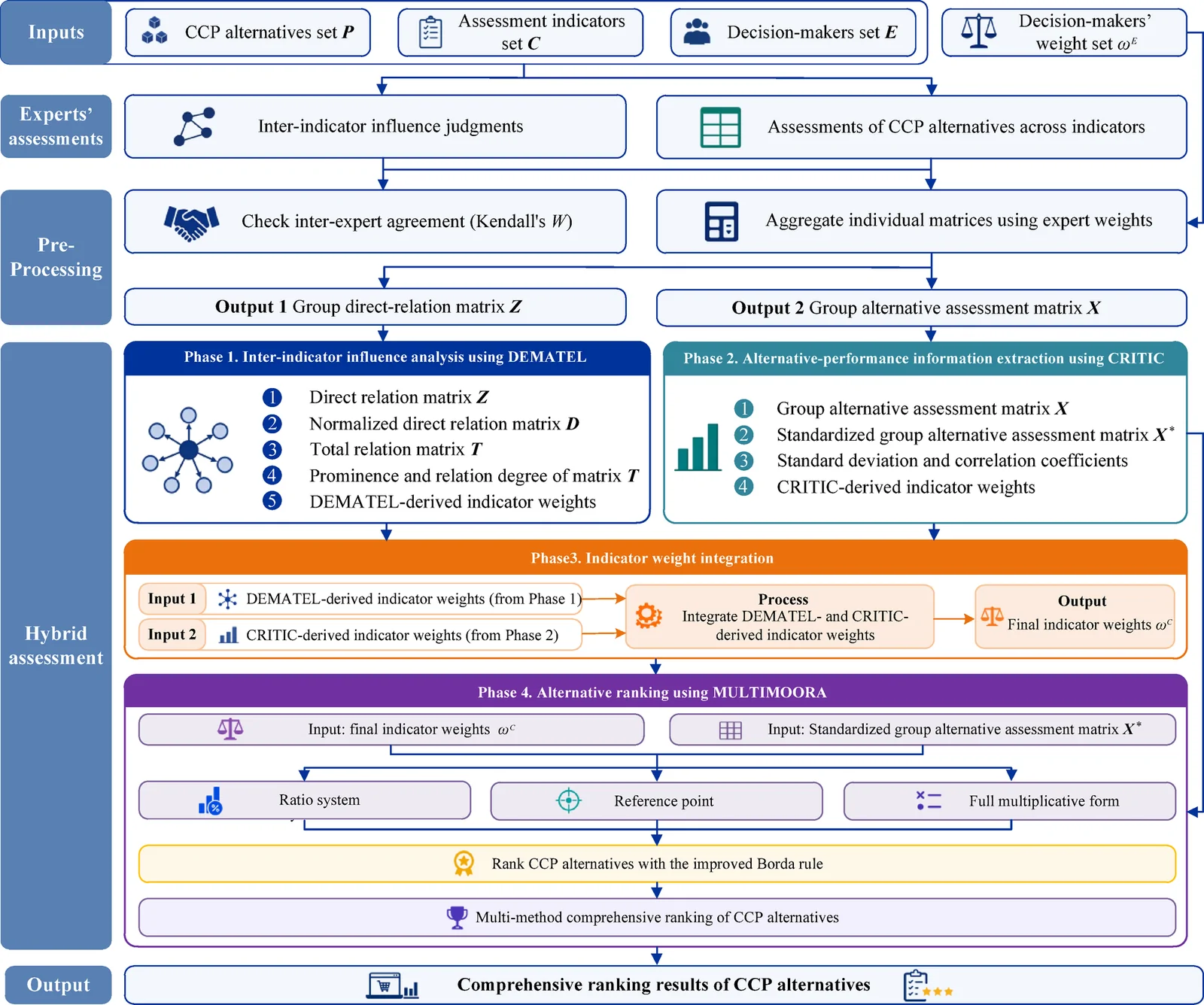
Implementation workflow and data relationships of the DEMATEL–CRITIC–MULTIMOORA hybrid assessment model.

Figure 2 illustrates the implementation workflow and data relationships of the DEMATEL–CRITIC–MULTIMOORA hybrid assessment model. Based on the given inputs, experts provide two types of assessments: inter-indicator influence judgments, and assessments of CCP alternatives across indicators. These raw assessments then undergo a pre-processing step: inter-expert agreement is checked using Kendall’s *W*, and individual matrices are aggregated using expert weights. This yields the group direct relation matrix ***Z*** (from the influence judgments) and the group alternative assessment matrix ***X*** (from the alternative assessments). Subsequently, the two processed information flows are used in parallel. The group direct relation matrix ***Z*** is fed into DEMATEL to produce DEMATEL-derived indicator weights. Meanwhile, the group alternative assessment matrix ***X*** is used in CRITIC to produce CRITIC-derived indicator weights; the same matrix ***X*** is also standardised to form the standardised group alternative assessment matrix X* for MULTIMOORA ranking. DEMATEL and CRITIC outputs are integrated to obtain the final indicator weights $\:{\omega\:}^{C}$. The final indicator weights $\:{\omega\:}^{C}$ and the standardised matrix X* are jointly adopted to implement the MULTIMOORA ranking.

The implementation process is organised into four phases that correspond to the functional roles of the three methods, as shown in Fig. 2: (1) inter-indicator influence analysis using DEMATEL, (2) alternative-performance information extraction using CRITIC, (3) indicator weight integration, and (4) alternative ranking using MULTIMOORA (via the ratio system, reference point approach, and improved Borda rule). The improved Borda rule synthesizes the outputs of the three MULTIMOORA sub-methods to obtain the multi-method comprehensive ranking of CCP alternatives, which is then delivered as the final comprehensive ranking results of CCP alternatives. For clarity, the notation used across these phases is defined as follows.

Let the set of CCP alternatives be denoted by $\:P=\left\{{P}_{1},{P}_{2},\dots\:,{P}_{m}\right\}$, where *m* is the number of alternatives to be evaluated. Let the set of assessment indicators be denoted by$\:\:C=\left\{{C}_{1}^{1},\dots\:,{C}_{R}^{1},\dots\:,{C}_{1}^{l},\dots\:,{C}_{R}^{l},\dots\:,{C}_{1}^{L},\dots\:,{C}_{R}^{L}\right\}$, and$\:{\:C}_{R}^{1}+{C}_{R}^{l}+{C}_{R}^{L}=n$, where *n* is the number of secondary indicators. Let the set of decision-makers be represented by $\:E=\left\{{E}_{1},{E}_{2},\dots\:,{E}_{k}\right\}$, and let the corresponding decision-maker weight vector be $\:{\omega\:}^{E}={\left({\omega\:}_{1},{\omega\:}_{2},\dots\:,{\omega\:}_{k}\right)}^{\mathrm{T}}$. The weight vector of the assessment indicators is denoted by $\:{\omega\:}^{C}={\left({\omega\:}_{1}^{1},\dots\:,{\omega\:}_{R}^{1},\dots\:,{\omega\:}_{1}^{l},\dots\:,{\omega\:}_{R}^{l},\dots\:,{\omega\:}_{1}^{L},\dots\:,{\omega\:}_{R}^{L}\right)}^{\mathrm{T}}$, where$\:{\:\omega\:}_{r}^{l}\ge\:0$, and $\:\sum\:_{l=1}^{1}\sum\:_{r=1}^{R}{\omega\:}_{r}^{l}=1$.

#### Inter-indicator influence analysis using DEMATEL

This subsection uses DEMATEL to extract inter-indicator influence information from expert judgments and convert it into DEMATEL-derived indicator weights.

Step 1. Establish the direct relation matrix ***Z***.

To measure the interrelationships among the *n* secondary indicators in the perceived cultural value assessment process for CCPs, *k* senior experts with extensive experience in product design and cultural studies are invited to assess the mutual influence relationships among the secondary indicators using a seven-point Likert scale. The judgment matrix ***Z***_*i*_ provided by expert *i* is given in Eq. (4).

4 $$\:{\boldsymbol{Z}}_{i}={\left({z}_{i\left(rs\right)}^{lu}\right)}_{n\times\:n}=\left[\begin{array}{ccccc}{z}_{i\left(11\right)}^{11}&\:\dots\:&\:{z}_{i\left(1r\right)}^{1l}&\:\dots\:&\:{z}_{i\left(1R\right)}^{1L}\\\: \vdots &\:\ddots\:&\: \vdots &\:\ddots\:&\: \vdots \\\:{z}_{i\left(r1\right)}^{l1}&\:\dots\:&\:{z}_{i\left(rr\right)}^{ll}&\:\dots\:&\:{z}_{i\left(rR\right)}^{lL}\\\: \vdots &\:\ddots\:&\: \vdots &\:\ddots\:&\: \vdots \\\:{z}_{i\left(R1\right)}^{L1}&\:\dots\:&\:{z}_{i\left(Rr\right)}^{Ll}&\:\dots\:&\:{z}_{i\left(RR\right)}^{LL}\end{array}\right]$$

where $\:{z}_{i\left(rs\right)}^{lu}$ denotes the score assigned by expert *i* to the influence of the *r*-th secondary indicator under the *l*-th primary indicator on the *s*-th secondary indicator under the *u*-th primary indicator.

Based on the expertise of each expert in CCP design and cultural studies, weights are assigned to the experts, denoted by $\:{\omega\:}_{i}^{E}$. The *k* individual judgment matrices are then aggregated to obtain the group direct relation matrix ***Z***, as shown in Eq. (5).

5 $$\:\boldsymbol{Z}={\left({z}_{rs}^{lu}\right)}_{n\times\:n}=\left[\begin{array}{ccccc}{z}_{11}^{11}&\:\dots\:&\:{z}_{1r}^{1L}&\:\dots\:&\:{z}_{1R}^{1L}\\\: \vdots &\:\ddots\:&\: \vdots &\:\ddots\:&\: \vdots \\\:{z}_{r1}^{l1}&\:\dots\:&\:{z}_{rr}^{ll}&\:\dots\:&\:{z}_{rR}^{lL}\\\: \vdots &\:\ddots\:&\: \vdots &\:\ddots\:&\: \vdots \\\:{z}_{R1}^{L1}&\:\dots\:&\:{z}_{Rr}^{Ll}&\:\dots\:&\:{z}_{RR}^{LL}\end{array}\right]$$

Step 2. Establish the normalized direct relation matrix ***D***.

Each element in the direct relation matrix ***Z*** is normalized to obtain the normalized direct relation matrix ***D***, as shown in Eq. (6).

6 $$\:\boldsymbol{D}={\left({d}_{rs}^{lu}\right)}_{n\times\:n}=\left[\begin{array}{ccccc}{d}_{11}^{11}&\:\dots\:&\:{d}_{1r}^{1l}&\:\dots\:&\:{d}_{1R}^{1L}\\\: \vdots &\:\ddots\:&\: \vdots &\: \vdots &\: \vdots \\\:{d}_{r1}^{l1}&\:\dots\:&\:{d}_{rr}^{ll}&\:\dots\:&\:{d}_{rR}^{lL}\\\: \vdots &\: \vdots &\: \vdots &\: \vdots &\: \vdots \\\:{d}_{R1}^{L1}&\:\dots\:&\:{d}_{Rr}^{Ll}&\:\dots\:&\:{d}_{RR}^{LL}\end{array}\right]$$

where $\:{d}_{rs}^{lu}$ is calculated by Eq. (7).

7 $$\:{d}_{rs}^{lu}=\frac{{z}_{rs}^{lu}}{\mathrm{m}\mathrm{a}{\mathrm{x}}_{\begin{array}{c}1\le\:l\le\:L\\\:1\le\:r\le\:R\end{array}}\sum\:_{u=1}^{L}\sum\:_{s=1}^{R}{z}_{rs}^{lu}}$$

Step 3. Obtain the total relation matrix ***T***.

Based on the normalized direct relation matrix ***D***, the total relation matrix ***T*** is derived using Eq. (8).

8 $$\:\boldsymbol{T}=\boldsymbol{D}(\boldsymbol{I}-\boldsymbol{D}{)}^{-1}$$

where ***I*** denotes the identity matrix.

Step 4. Calculate the row sum and column sum of the total relation matrix ***T***.

Let $\:{A}_{r}^{l}$ and $\:{B}_{r}^{l}$ denote the row sum and column sum of the total relation matrix ***T***, representing the influence degree and the influenced degree of the *r*-th secondary indicator under the *l*-th primary indicator, respectively. Accordingly, $\:{A}_{r}^{l}$+$\:{B}_{r}^{l}$and $\:{A}_{r}^{l}$−$\:{B}_{r}^{l}$represent its prominence and relation degree. The former indicates overall importance, whereas the latter embodies the strength and direction of its causal relationship with other indicators.

9 $$\:{A}_{r}^{l}=\sum\:_{u=1}^{L}\sum\:_{s=1}^{R}{t}_{rs}^{lu}\hspace{1em}l=\mathrm{1,2},\dots\:,L;r=\mathrm{1,2},\dots\:,R$$

10 $$\:{B}_{r}^{l}=\sum\:_{u=1}^{L}\sum\:_{s=1}^{R}{t}_{sr}^{ul}\hspace{1em}l=\mathrm{1,2},\dots\:,L;r=\mathrm{1,2},\dots\:,R$$

11 $$\:{A}_{r}^{l}+{B}_{r}^{l}=\sum\:_{u=1}^{L}\sum\:_{s=1}^{R}{t}_{rs}^{lu}+\sum\:_{u=1}^{L}\sum\:_{s=1}^{R}{t}_{sr}^{ul}\hspace{1em}l=\mathrm{1,2},\dots\:,L;r=\mathrm{1,2},\dots\:,R$$

12 $$\:{A}_{r}^{l}-{B}_{r}^{l}=\sum\:_{u=1}^{L}\sum\:_{s=1}^{R}{t}_{rs}^{lu}-\sum\:_{u=1}^{L}\sum\:_{s=1}^{R}{t}_{sr}^{ul}\hspace{1em}l=\mathrm{1,2},\dots\:,L;r=\mathrm{1,2},\dots\:,R$$

Step 5. Determine the DEMATEL-derived indicator weights.

Based on Eqs. (11) and (12), the DEMATEL-derived weight of each secondary indicator is obtained using Eq. (13). In this transformation, prominence reflects the overall connectedness of an indicator in the influence system, whereas relation degree reflects whether the indicator tends to act as a driver or a receiver. Equation (13) combines these two structural attributes and normalizes them so that the resulting DEMATEL-derived weights sum to one.

13 $$\:{\left({\omega\:}_{r}^{l}\right)}^{s}=\frac{\sqrt{{({A}_{r}^{l}+{B}_{r}^{l})}^{2}+{({A}_{r}^{l}-{B}_{r}^{l})}^{2}}}{{\sum\:}_{l=1}^{L}\sum\:_{r=1}^{R}\sqrt{{({A}_{r}^{l}+{B}_{r}^{l})}^{2}+{({A}_{r}^{l}-{B}_{r}^{l})}^{2}}}$$

#### Alternative-performance information extraction using CRITIC

This subsection uses CRITIC to extract alternative-performance information from the group alternative assessment matrix and convert it into CRITIC-derived indicator weights.

Step 1. Construct group alternative assessment matrix ***X***.

Let the assessment matrix ***X***_*i*_ of the *i*-th expert for the *n* assessment indicators of the *m* CCPs be denoted as shown in Eq. (14).

14 $$\:{\boldsymbol{X}}_{i}={\left({x}_{i\left(r\right)}^{p\left(l\right)}\right)}_{m\times\:n}=\left[\begin{array}{ccccc}{x}_{i\left(1\right)}^{1\left(1\right)}&\:\dots\:&\:{x}_{i\left(r\right)}^{1\left(l\right)}&\:\dots\:&\:{x}_{i\left(R\right)}^{1\left(L\right)}\\\: \vdots &\:\ddots\:&\: \vdots &\: \vdots &\: \vdots \\\:{x}_{i\left(1\right)}^{p\left(1\right)}&\:\dots\:&\:{x}_{i\left(r\right)}^{p\left(l\right)}&\:\dots\:&\:{x}_{i\left(R\right)}^{p\left(L\right)}\\\: \vdots &\:\ddots\:&\: \vdots &\: \vdots &\: \vdots \\\:{x}_{i\left(1\right)}^{m\left(1\right)}&\:\dots\:&\:{x}_{i\left(r\right)}^{m\left(l\right)}&\:\dots\:&\:{x}_{i\left(R\right)}^{m\left(L\right)}\end{array}\right]$$

where $\:{x}_{i\left(r\right)}^{p\left(l\right)}$represents the assessment value assigned by the *i*-th expert to the *r*-th secondary indicator under the *l*-th primary indicator for the *p*-th CCP.

Following the same approach as described in Sect.  4.2.1, weights are assigned to each expert based on their expertise in CCP design and cultural studies, denoted as $\:{\omega\:}_{i}^{E}$. The initial alternative assessment matrices of the *k* senior experts are then aggregated to obtain the group alternative assessment matrix $\:\boldsymbol{X}={\left({x}_{\left(r\right)}^{p\left(l\right)}\right)}_{m\times\:n}$.

Step 2. Establish the standardized group alternative assessment matrix X^*^.

Since the CRITIC method is used in the weighting stage and the MULTIMOORA method is used in the ranking stage, this study adopts vector normalization to ensure scale consistency between the two stages. The standardized group alternative assessment matrix X^*^ is obtained using Eq. (15). This normalization removes dimensional differences while preserving proportional relationships among indicators and avoids the zero-value problem that may arise from min–max normalization and distort multiplicative calculations.

15 $$\:{\left({x}_{\left(r\right)}^{p\left(l\right)}\right)}^{\ast\:}=\frac{\left({x}_{\left(r\right)}^{p\left(l\right)}\right)}{\sqrt{{\sum\:}_{p=1}^{m}{\left({x}_{\left(r\right)}^{p\left(l\right)}\right)}^{2}}}$$

Step 3. Calculate standard deviations and correlation coefficients.

Based on the standardized group alternative assessment matrix X^*^, the standard deviation and correlation coefficient of each secondary indicator are obtained using Eqs. (16) and (17), respectively.

16 $$\:{\sigma\:}_{r}^{l}=\sqrt{\frac{1}{m-1}{\sum\:}_{p=1}^{m}{\left({\left({x}_{\left(r\right)}^{p\left(l\right)}\right)}^{\ast\:}-\overline{{\left({x}_{\left(r\right)}^{\left(l\right)}\right)}^{\ast\:}}\right)}^{2}}$$

17 $$\:{\gamma\:}_{rs}^{lu}=cov\left({\left({X}_{r}^{l}\right)}^{\ast\:},{\left({X}_{s}^{u}\right)}^{\ast\:}\right)/\left({\sigma\:}_{r}^{l}{\sigma\:}_{s}^{u}\right)\:\:\:\:\:\:\:l,u=\mathrm{1,2},\dots\:,L;r,s=\mathrm{1,2},\dots\:,R$$

where $\:{\left({X}_{r}^{l}\right)}^{\ast\:}$and $\:{\left({X}_{s}^{u}\right)}^{\ast\:}$represent the (*l*-1)*R* + *r*-th and (*u*-1)*R* + *s*-th columns of X^*^, respectively.

Step 4. Calculate the CRITIC-derived indicator weights.

In the CRITIC method, an indicator receives a larger weight when it shows greater variation across alternatives and lower redundancy with other indicators. In this study, the CRITIC-derived weight represents a relative weight extracted from the group alternative assessment matrix, rather than an absolute measure of objectivity. The amount of information contained in the *r*-th secondary indicator under the *l*-th primary indicator is calculated using Eq. (18). A larger value of $\:{E}_{r}^{l}$ indicates that the indicator contains more information. The corresponding CRITIC-derived weight is then obtained using Eq. (19). Accordingly, the greater the amount of information provided by an indicator, the larger its weight.

18 $$\:{E}_{r}^{l}={\sigma\:}_{r}^{l}{\sum\:}_{u=1}^{L}{\sum\:}_{s=1}^{R}\left(1-{\gamma\:}_{rs}^{lu}\right)$$

19 $$\:{\left({\omega\:}_{r}^{l}\right)}^{o}=\frac{{E}_{r}^{l}}{\sum\:_{l=1}^{L}\sum\:_{r=1}^{R}{E}_{r}^{l}}$$

#### Indicator weight integration

To integrate the indicator influence information obtained from DEMATEL and the alternative-performance information obtained from CRITIC, this study adopts a linear weighting approach, as shown in Eq. (20). Here, *ϕ* and *τ* represent the contribution coefficients of the DEMATEL-derived and CRITIC-derived weights, respectively, subject to the constraint *ϕ* + *τ* = 1. The values of *ϕ* and *τ* can be adjusted according to the decision context.

20 $$\:{\omega\:}_{r}^{l}=\phi\:{\left({\omega\:}_{r}^{l}\right)}^{s}+\tau\:{\left({\omega\:}_{r}^{l}\right)}^{o}$$

#### Alternative ranking using MULTIMOORA

Based on the standardized group alternative assessment matrix and the final indicator weights, MULTIMOORA is used to rank CCP alternatives through three complementary aggregation procedures: the ratio system, the reference point approach, and the full multiplicative form. These procedures compare alternatives from different perspectives of weighted performance, distance from the reference point, and multiplicative utility, thereby reducing dependence on a single ranking rule. The sub-rankings obtained from the three procedures are then aggregated using an improved Borda rule to generate the comprehensive ranking of CCP alternatives.

Step 1. Ratio system (RS).

Based on the standardized group alternative decision matrix X^*^, the integrated utility values of the benefit-type and cost-type indicators are first obtained by combining the normalized values with the corresponding indicator weights. The integrated ratio value of each CCP is then calculated using Eq. (21). The CCPs are subsequently ranked in descending order of *χ*_*p*_, which can be represented as: $\:{R}_{RS}=\left\{{P}_{p}\mid\:\mathrm{max}{\chi\:}_{p}>\dots\:>{P}_{p}\mid\:\mathrm{min}{\chi\:}_{p}\right\}$ .

21 $$\:{\chi\:}_{p}={\sum\:}_{l=1}^{L}{\sum\:}_{r=1}^{g}{\omega\:}_{r}^{l}{\left({x}_{\left(r\right)}^{p\left(l\right)}\right)}^{\ast\:}-{\sum\:}_{l=1}^{L}{\sum\:}_{r=g+1}^{R}{\omega\:}_{r}^{l}{\left({x}_{\left(r\right)}^{p\left(l\right)}\right)}^{\ast\:}$$

where $\:{\omega\:}_{r}^{l}(l=1,\dots\:,L;r=1,\dots\:,R)$ represents the weight of the *r*-th secondary indicator under the *l*-th primary indicator. *g* is the number of benefit-type indicators, and *R* - *g* is the number of cost-type indicators under the *l*-th primary indicator.

Step 2. Reference point (RP).

Based on the normalized group alternative decision matrix X^*^, the comprehensive reference point $\:{r}_{r}^{l}$ can be defined as:

22 $$\:{r}_{r}^{l}=\left\{\begin{array}{cc}{\mathrm{m}\mathrm{a}\mathrm{x}}_{p}{\left({x}_{\left(r\right)}^{p\left(l\right)}\right)}^{\ast\:},&\:r\le\:g,l=1,\dots\:,L\\\:{\mathrm{m}\mathrm{i}\mathrm{n}}_{p}{\left({x}_{\left(r\right)}^{p\left(l\right)}\right)}^{\ast\:},&\:r>g,l=1,\dots\:,L\end{array}\right.$$

where *r* ≤ *g* denotes that $\:{C}_{r\:}^{l}$ is a benefit-type indicator, *r* > *g* denotes that $\:{C}_{r\:}^{l}$ is a cost-type indicator.

The weighted distance between *P*_*p*_ and the comprehensive reference point can be calculated using Eq. (23). Then, the CCPs are ranked in ascending order of *δ*_*p*_ (denoted as $\:{R}_{RP}=\left\{{P}_{p}\mid\:\mathrm{m}\mathrm{in}{\delta\:}_{p}>\dots\:>{P}_{p}\mid\:\mathrm{m}\mathrm{ax}{\delta\:}_{p}\right\}$), with smaller values indicating better performance.

23 $$\:{\delta\:}_{p}=\underset{\begin{array}{c}1\le\:l\le\:L\\\:1\le\:r\le\:R\end{array}}{\mathrm{max}}{\omega\:}_{r}^{l}\left|{r}_{r}^{l}-{\left({x}_{\left(r\right)}^{p\left(l\right)}\right)}^{\ast\:}\right|$$

Step 3. Full multiplicative form (FMF).

The geometric weighted aggregation operator is used to derive the utility values for the benefit and cost indicators, respectively. Subsequently, the FMF utility value is computed using Eq. (24). Next, the CCPs are ranked in descending order of *φ*_*p*_, which can be expressed as: $\:{R}_{FMF}=\left\{{P}_{p}\mid\:\mathrm{m}\mathrm{ax}{\varphi\:}_{p}>\dots\:>{P}_{p}\mid\:\mathrm{m}\mathrm{im}{\varphi\:}_{p}\right\}$.

24 $$\:{\varphi\:}_{p}={\prod\:}_{l=1}^{L}{\prod\:}_{r=1}^{g}{\left({\left({x}_{\left(r\right)}^{p\left(l\right)}\right)}^{\ast\:}\right)}^{{\omega\:}_{r}^{l}}/{\prod\:}_{l=1}^{L}{\prod\:}_{r=g+1}^{R}{\left({\left({x}_{\left(r\right)}^{p\left(l\right)}\right)}^{\ast\:}\right)}^{{\omega\:}_{r}^{l}}$$

Step 4. Rank CCPs with the improved Borda rule.

Because the three MULTIMOORA sub-models generate utility values with different scales and ranking directions, the utilities are first normalized before final aggregation. The improved Borda rule is then used to combine both the normalized utility information and the rank-order information from the RS, RP, and FMF methods, so that the final ranking is not determined by a single aggregation perspective. Let $\:{\chi\:}_{p}^{\ast\:}$, $\:{\delta\:}_{p}^{\ast\:}$ and $\:{\varphi\:}_{p}^{\ast\:}$ denote the normalized utility values obtained from the RS, RP and FMF methods, respectively. Here, $\:{\chi\:}_{p}^{\ast\:}$ can be calculated by Eq. (25), and the same normalization is applied to $\:{\delta\:}_{p}^{\ast\:}$ and $\:{\varphi\:}_{p}^{\ast\:}$. The overall ranking score of *P*_*p*_ is then calculated by improved Borda rule with Eq. (26).

25 $$\:{\chi\:}_{p}^{\ast\:}=\frac{{\chi\:}_{p}}{\sqrt{{\sum\:}_{p=1}^{m}{\left({\chi\:}_{p}\right)}^{2}}}$$

26 $$\:IB{S}_{p}={\chi\:}_{p}^{\ast\:}\times\:\frac{m-r\left({\chi\:}_{p}\right)+1}{m(m+1)/2}-{\delta\:}_{p}^{\ast\:}\times\:\frac{r\left({\delta\:}_{p}\right)}{m(m+1)/2}+{\varphi\:}_{p}^{\ast\:}\times\:\frac{m-r\left({\varphi\:}_{p}\right)+1}{m(m+1)/2}\:\:\:\:p=\mathrm{1,2},\dots\:m$$

where *r*(*χ*_*p*_), *r*(*δ*_*p*_), and *r*(*φ*_*p*_) are the priority orders of the RS, RP and FMF approaches, respectively.

### Ethical approval and informed consent

All procedures involving human participants in this study, including expert assessment and the questionnaire survey, were conducted in accordance with the relevant institutional guidelines and regulations of Xi’an Technological University and the ethical principles of the Declaration of Helsinki where applicable. The study protocol was reviewed by the Academic Committee of the School of Design, Xi’an Technological University, which is responsible for research ethics and integrity review within the School. The Committee determined that the study was exempt from full ethical review because it involved minimal-risk social survey research, including anonymous questionnaire responses and expert assessments, without biomedical intervention, deception, or collection of personally identifiable private information. Informed consent was obtained from all participants before participation. For the anonymous questionnaire survey, the Committee approved a waiver of signed informed consent. An information sheet was presented at the beginning of the questionnaire, explaining the purpose of the study, the voluntary nature of participation, the anonymity of responses, and participants’ right to discontinue participation before submission without consequence. Completion and submission of the questionnaire were regarded as voluntary informed consent. No personally identifiable information was collected. For the expert assessment, all experts provided informed consent after being informed of the study purpose, assessment procedure, and use of the results. Expert responses were used only for research purposes and reported in aggregated form. Personal identifiers were removed during data processing, and no individual-level identifiable information is disclosed in this study.

## Case application

### Selection of product cases

To examine the applicability of the proposed perceived cultural value assessment approach in a controlled e-commerce visual-textual context, this study selected cultural aromatherapy burners from e-commerce platforms as the case objects. The selection was based on three considerations: First, aromatherapy burners combine strong visual expressiveness, clear context-dependent use, and rich cultural symbolism, allowing a relatively comprehensive correspondence with the three-domain assessment framework of formal representation, usage inference, and meaning construction. Second, this product category has high visibility in the online CCP market, which facilitates the collection of relatively stable visual-textual materials under comparable platform conditions. Third, compared with commemorative ornaments with limited functional cues or conventional crafts dominated by decorative attributes, aromatherapy burners exhibit a clearer relationship between use-related cues and cultural meaning, making them suitable for applying and illustrating the proposed perceived cultural value assessment method.

In the sampling process, Taobao was used as the data source, and the keyword “cultural aromatherapy burners” was employed for retrieval. The following criteria were applied:

1. The main product image and detail page were both accessible;
2. The visual-textual information supported judgment on at least two assessment domains;
3. The cultural theme of the product was explicitly articulated;
4. Samples with co-branding, strong promotional packaging, or severely missing information were excluded.

Based on these criteria, six representative products were selected as the alternatives for assessment, as shown in Table 3. To reduce the potential influence of differences in stimulus presentation, all case materials were standardized before assessment. Specifically, the backgrounds of all product images were removed and the images were presented at a unified scale to control for variations in page environment and photographic style. In addition, based on the original visual-textual information provided on the platform, a descriptive text of approximately 100 Chinese characters was prepared for each product using a unified template. Only information directly related to formal representation, usage inference, and meaning construction was retained, without adding further interpretive content. The processed materials served as the unified stimulus set for the subsequent expert assessment and user-side questionnaire survey. All standardized visual stimuli were sourced from publicly available product pages on Taobao and only used for internal expert and user assessment without publication.

**Table 3:** Visual-textual information of cultural aromatherapy burners.

	Product image	Product introduction
*P*_1_		Inspired by an armored auspicious tortoise, this product presents a dynamic yet dignified form with a lowered posture, backward-looking profile, and surrounding shell patterns. It serves as an aromatherapy ornament for reading, tea drinking, meditation, and short rest. The design symbolizes protection, longevity, and enduring good fortune.
*P*_2_		This lotus-themed product combines openwork floral patterns with layered petals, creating a refined and elegant form. It functions as an aromatherapy ornament for home and office display. The design conveys auspiciousness, purity, and graceful elegance.
*P*_3_		Drawing on Auspicious Cranes, this product integrates crane imagery with three ruyi-shaped feet in a lively and elegant form. It serves as an aromatherapy ornament for home and office settings. The design conveys an auspicious meaning.
*P*_4_		Inspired by Listening to the Qin, this product features a smooth vessel body, a stable three-legged base, and simplified auspicious ornamentation with bat motifs. It integrates fragrance diffusion, deodorizing, sleep accompaniment, and night-light functions. The design expresses wishes for auspiciousness, prosperity, and peace.
*P*_5_		This product reinterprets the Warring States Bronze Dou with Inlaid Turquoise and Coiled Chi Pattern, preserving its round body, ring handles, tall stem, and decorative bands in a solid and finely crafted form. It functions as an aromatherapy object for office, reception, and rest settings. The design conveys cultural inheritance, refined living, and everyday aesthetics.
*P*_6_		Centered on a museum signature treasure, this product adopts a duck motif and recreates the texture and formal character of the original artifact in a calm and elegant style. It functions as an essential-oil diffuser for home and office display. The design symbolizes scholarly success, distinction, and a promising future.

Photographs displayed in this table were independently captured by the authors to intuitively display the overall physical appearance of each product for readers’ reference. All standardized visual stimuli adopted in expert assessment and user questionnaires were background-removed product images sourced from Taobao, rather than the photos shown herein.

### Application of the hybrid assessment model

Based on the perceived cultural value assessment indicators established in Sect.  3, this study invited five experts, selected from the eight experts who had participated in the content validity review, to form an expert panel for indicator weighting and product case assessment. These experts had extensive experience in design, cultural communication, and user research, and their prior involvement in the validity assessment helped ensure familiarity with the perceived cultural value indicators, their evaluative boundaries, and the corresponding observable evidence domains. All evaluations were conducted using a 7-point Likert scale (1 = strongly disagree, 7 = strongly agree). The implementation process is described as follows.

#### Indicator weighting results

##### DEMATEL-derived indicator weights

In the DEMATEL phase, the five experts assessed the inter-indicator influence relationships among the 12 secondary indicators using a seven-point Likert scale. The raw expert judgment matrices are reported in Supplementary Table S1, including expert ID, source indicator, target indicator, and corresponding score. Before aggregating the individual judgments, Kendall’s coefficient of concordance (Kendall’s *W*) was used to examine the consistency of experts’ indicator-importance rankings. Since DEMATEL-derived weights reflect each indicator’s structural position within the inter-indicator influence system, the rankings used for the concordance test were determined based on the DEMATEL-derived weights calculated separately for each expert. The results were *W* = 0.128, *χ*^2^ = 7.031, *df* = 11, and *p* = 0.797, indicating evident heterogeneity among experts regarding the relative importance of the indicators.

Given that perceived cultural value assessment involves multiple aspects, including design expression, usage understanding, and cultural meaning interpretation, such heterogeneity in expert judgments is considered reasonable. To form a group assessment result while retaining heterogeneous expert judgments, expert weights were assigned according to each expert’s research experience and representative academic achievements in the relevant fields, yielding $\:{\omega\:}^{E}={\left(\mathrm{0.15,0.2,0.25,0.18,0.22}\right)}^{\mathrm{T}}$. On this basis, the individual DEMATEL judgment matrices were aggregated using the assigned expert weights to obtain the group direct relation matrix ***Z***. Following Eqs. (6)–(13), SPSSAU was used to calculate the normalized direct relation matrix ***D***, the total relation matrix ***T***, influence degree, influenced degree, prominence, relation degree, and DEMATEL-derived indicator weights. The group direct relation matrix ***Z***, normalized direct relation matrix ***D***, total relation matrix ***T*** are provided in Supplementary Tables S2–S4. The key outputs of the DEMATEL phase are reported in Table 4.

**Table 4:** Influence degree, influenced degree, prominence, relation degree, and DEMATEL-derived weights of each indicator.

	$\:{C}_{1}^{1}$	$\:{C}_{2}^{1}$	$\:{C}_{3}^{1}$	$\:{C}_{4}^{1}$	$\:{C}_{1}^{2}$	$\:{C}_{2}^{2}$	$\:{C}_{3}^{2}$	$\:{C}_{4}^{2}$	$\:{C}_{1}^{3}$	$\:{C}_{2}^{3}$	$\:{C}_{3}^{3}$	$\:{C}_{4}^{3}$
Influence degree	11.639	11.306	10.903	10.805	9.906	9.479	10.188	11.162	10.590	10.803	10.528	11.627
Influenced degree	10.885	10.991	10.654	10.412	10.193	10.300	11.067	10.744	11.148	11.427	10.546	10.571
Prominence	22.525	22.296	21.557	21.217	20.099	19.779	21.255	21.906	21.738	22.230	21.074	22.198
Relation degree	0.754	0.315	0.248	0.394	-0.286	-0.821	-0.879	0.418	-0.557	-0.624	-0.018	1.057
$\:{\left({\omega\:}_{r}^{l}\right)}^{s}$	0.0873	0.0865	0.0836	0.0823	0.0779	0.0767	0.0824	0.0849	0.0843	0.0862	0.0817	0.0861

##### CRITIC-derived indicator weights

In the CRITIC phase, the same five experts assessed the performance of the six cultural aromatherapy burner alternatives against the 12 perceived cultural value indicators using a seven-point Likert scale. The raw expert alternative assessment matrices are reported in Supplementary Table S5, including expert ID, product alternative, assessment indicator, and corresponding score. Before aggregating the individual alternative assessment matrices, Kendall’s *W* was used to examine the consistency of experts’ rankings of the six alternatives. The rankings were determined based on each expert’s overall scores of the alternatives across the 12 indicators. Because tied ranks occurred, the tie-corrected Kendall’s *W* was adopted. The results were *W* = 0.414, *χ*^2^ = 10.353, *df* = 5, and *p* = 0.066, indicating a certain degree of coordination among the experts, although the agreement was not statistically significant at the 0.05 level.

Based on the assigned expert weights $\:{\omega\:}^{E}={\left(\mathrm{0.15,0.2,0.25,0.18,0.22}\right)}^{\mathrm{T}}$, five alternative assessment matrices were aggregated to obtain the group alternative assessment matrix ***X***. Following Eqs. (15)–(19), SPSSAU was used to calculate the standardized group alternative assessment matrix X^*^, standard deviations of the indicators, inter-indicator correlation coefficients, information content, and CRITIC-derived indicator weights. The group alternative assessment matrix ***X***, and standardized group alternative assessment matrix X^*^ are provided in Supplementary Tables S6–S7; the key outputs of the CRITIC phase are reported in Table 5.

**Table 5:** CRITIC-derived weights of each indicator.

	$\:{C}_{1}^{1}$	$\:{C}_{2}^{1}$	$\:{C}_{3}^{1}$	$\:{C}_{4}^{1}$	$\:{C}_{1}^{2}$	$\:{C}_{2}^{2}$	$\:{C}_{3}^{2}$	$\:{C}_{4}^{2}$	$\:{C}_{1}^{3}$	$\:{C}_{2}^{3}$	$\:{C}_{3}^{3}$	$\:{C}_{4}^{3}$
$\:{E}_{r}^{l}$	0.294	0.315	0.114	0.217	0.203	0.240	0.228	0.212	0.180	0.244	0.258	0.351
$\:{\left({\omega\:}_{r}^{l}\right)}^{o}$	0.1029	0.1102	0.0400	0.0759	0.0712	0.0841	0.0798	0.0742	0.0632	0.0853	0.0902	0.1230

##### Calculation of combined indicator weights

The DEMATEL-derived weights and CRITIC-derived weights reflect different aspects of the indicator-weight determination process. The former captures inter-indicator influence information extracted from expert judgments, whereas the latter captures alternative-performance information derived from the group alternative assessment matrix. To avoid assigning prior preference to either source of information, the contribution coefficients of the two components were both set to 0.5 in this study. The final indicator weights were then calculated using Eq. (20), and the results are reported in Table 6. These weights were used in the subsequent MULTIMOORA-based ranking of CCP alternatives.

**Table 6:** Final indicator weights.

	$\:{C}_{1}^{1}$	$\:{C}_{2}^{1}$	$\:{C}_{3}^{1}$	$\:{C}_{4}^{1}$	$\:{C}_{1}^{2}$	$\:{C}_{2}^{2}$	$\:{C}_{3}^{2}$	$\:{C}_{4}^{2}$	$\:{C}_{1}^{3}$	$\:{C}_{2}^{3}$	$\:{C}_{3}^{3}$	$\:{C}_{4}^{3}$
$\:{\omega\:}_{r}^{l}$	0.0951	0.0984	0.0618	0.0791	0.0746	0.0804	0.0811	0.0796	0.0738	0.0858	0.0860	0.1046

#### Ranking results of CCP alternatives

The perceived cultural value ranking of CCP alternatives was conducted using MULTIMOORA. In the case application, all indicators were treated as benefit-type indicators. Based on the standardized group alternative assessment matrix X^*^ and the final indicator weights obtained above, the original utility values of each alternative were calculated using the ratio system, the reference point method, and the full multiplicative form according to Eqs. (21), (23), and (24), respectively. These original utility values are reported in Supplementary Table S8. To make the outputs of the three sub-models comparable, the utility values were then normalized using Eq. (25). The normalized utility values and the final comprehensive ranking obtained using the improved Borda rule according to Eq. (26) are presented in Table 7.

Overall, the ranking results under the three sub-models are generally consistent. The ratio system and full multiplicative form produce the same ranking order, while the reference point method identifies the same top-three alternatives but with a different internal order. The final comprehensive ranking is *P*_3_ > *P*_2_>*P*_4_ > *P*_6_>*P*_1_>*P*_5_. Specifically, *P*_3_ ranks first in both the ratio system and the full multiplicative form and ranks third under the reference point method; nevertheless, given its consistent top performance across two models, it can be regarded as the best-performing alternative in terms of overall perceived cultural value within the current sample set. *P*_2_ and *P*_4_ also show relatively strong performance and constitute the second tier of alternatives. By contrast, *P*_5_ ranks consistently lower across the three sub-models, indicating comparatively weaker coordination among formal representation, usage inference, and meaning construction.

**Table 7:** Normalized utility values and rankings under the three MULTIMOORA sub-models and final ranking.

	Ratio system		Reference point		Full multiplicative form		The final ranking
	$\:{\chi\:}_{p}^{\ast\:}$	*r*(χ_*p*_)	$\:{\delta\:}_{p}^{\ast\:}$	*r*(δ_*p*_)	$\:{\varphi\:}_{p}^{\ast\:}$	*r*(φ_*p*_)	IBS_*p*_
*P*_1_	0.4021	5	0.4794	5	0.4022	5	5
*P*_2_	0.4187	2	0.2782	1	0.4186	2	2
*P*_3_	0.4312	1	0.3410	3	0.4314	1	1
*P*_4_	0.4086	3	0.3375	2	0.4087	3	3
*P*_5_	0.3820	6	0.4975	6	0.3818	6	6
*P*_6_	0.4053	4	0.4639	4	0.4051	4	4

### Practical implications

This study provides an operational tool for the preliminary screening and comparison of CCPs under e-commerce visual-textual presentation. Compared with conventional approaches that rely mainly on experience and aesthetic preference, the proposed three-domain perceived cultural value framework and the DEMATEL–CRITIC–MULTIMOORA assessment model translate cultural semantic expression into structured indicators, evidence domains, and ranking results, thereby offering a more transparent reference for product analysis and design decision-making in platform-based consumption contexts.

Its practical value is reflected in three aspects. First, at the concept screening stage, the method can be used to compare alternative design schemes in terms of perceived cultural value and identify more promising directions for further development. Second, at the competitor analysis stage, it converts the visual-textual presentation of different products on e-commerce platforms into comparable assessment objects, thus supporting product positioning and differentiation analysis. Third, at the page optimization stage, the evidence domains established in this study can serve as diagnostic references for identifying omissions and inconsistencies in the formal expression, usage-related communication, and meaning delivery of cultural cues.

## Results and discussion

To further examine the applicability and stability of the proposed method, two comparative analyses were conducted. First, a sensitivity analysis of the DEMATEL–CRITIC contribution coefficient was performed to assess the stability of ranking results under different weight-integration settings. Second, a user-side comparison based on questionnaire data was carried out to compare model outputs with user-side assessment results.

### Sensitivity analysis of the DEMATEL–CRITIC contribution coefficient

In MADM problems, the allocation ratio between DEMATEL-derived weights and CRITIC-derived weights can alter the final alternative ranking. To examine the stability of the ranking results under different weight-integration settings, five coefficient schemes were designed to reflect three practical decision tendencies: balanced integration, greater emphasis on alternative-performance information, and greater emphasis on inter-indicator influence information. Five sets of weight allocation combinations are comparatively analyzed, as shown in Table 8.

**Table 8:** Sensitivity analysis under different DEMATEL–CRITIC contribution coefficients.

	*ϕ*	*τ*	Ranking
Balanced integration scheme	0.5	0.5	*P*_3_>*P*_2_>*P*_4_>*P*_6_>*P*_1_>*P*_5_
Alternative-performance emphasis scheme 1	0.3	0.7	*P*_3_>*P*_2_>*P*_4_>*P*_6_>*P*_1_>*P*_5_
Alternative-performance emphasis scheme 2	0.4	0.6	*P*_3_>*P*_2_>*P*_4_>*P*_6_>*P*_1_>*P*_5_
Inter-indicator influence emphasis scheme 1	0.6	0.4	*P*_3_>*P*_2_>*P*_4_>*P*_6_>*P*_1_>*P*_5_
Inter-indicator influence emphasis scheme 2	0.7	0.3	*P*_3_>*P*_2_>*P*_4_>*P*_6_>*P*_1_>*P*_5_

*ϕ* denotes the contribution coefficient of the DEMATEL-derived weights, and *τ* denotes the contribution coefficient of the CRITIC-derived weights, satisfying *ϕ* + *τ* = 1.

As shown in Table 8, changes in the weighting coefficients did not alter the ranking results. Under all five combinations, the six products retained the same order, indicating that the ranking result is stable under the tested coefficient settings. The selected coefficient schemes cover balanced integration and two directional deviations toward either alternative-performance information or inter-indicator influence information. Accordingly, *ϕ* = 0.5 and *τ* = 0.5 were adopted as the baseline setting. This balanced integration setting gives equal contribution to DEMATEL-derived and CRITIC-derived weights, and sensitivity results suggest that the ranking outputs remain stable within the tested coefficient range.

### User-side comparison based on questionnaire data

In this study, “external evidence” refers to user-side questionnaire assessment data collected independently from the expert-based DEMATEL–CRITIC–MULTIMOORA calculation process. It does not refer to real-world sales data, platform behavioral data such as clicks or conversion rates, or assessments from another expert panel. The purpose of introducing this evidence is to examine whether the product ranking generated by the expert-based model is broadly consistent with non-expert users’ perceived cultural value assessments under the same e-commerce visual-textual materials. Because the questionnaire data were collected outside the expert assessment process, they offer an external reference for interpreting the model outputs and partly reduce the risk that the results only reflect the assumptions of the overlapping expert panel. However, they cannot fully eliminate confirmation bias or validate actual market performance.

Accordingly, a questionnaire was designed to collect user-side perceived cultural value assessments for the six products. In the questionnaire, each product was presented using a standardized product image and an approximately 100-word description organized around the three primary dimensions of formal representation, usage inference, and meaning construction. To support non-expert interpretation, each secondary indicator was accompanied by a concise explanation rather than being presented only as a technical term. Because asking respondents to evaluate all 6 products × 12 indicators in a single questionnaire would substantially increase response burden^49^, a balanced incomplete block design (BIBD) was adopted to distribute product evaluations across questionnaire packages^50^. With six products, three products per questionnaire package, and ten unordered product combinations (Table 9), the design reduced individual burden while maintaining relatively balanced exposure. A total of 300 questionnaires were collected, of which 240 valid responses were retained after data cleaning. Each product ultimately received 120 valid evaluations, forming a respondent–product long-format dataset for the user-side comparison.

**Table 9:** BIBD-based product combinations.

No.	Portfolio	No.	Portfolio	No.	Portfolio	No.	Portfolio	No.	Portfolio
1	*P*_1_ *P*_2_ *P*_3_	2	*P*_1_ *P*_2_ *P*_4_	3	*P*_1_ *P*_3_ *P*_5_	4	*P*_1_ *P*_4_ *P*_6_	5	*P*_1_ *P*_5_ *P*_6_
6	*P*_2_ *P*_3_ *P*_6_	7	*P*_2_ *P*_4_ *P*_5_	8	*P*_2_ *P*_5_ *P*_6_	9	*P*_3_ *P*_4_ *P*_5_	10	*P*_3_ *P*_4_ *P*_6_

Before ranking comparison, a confirmatory factor analysis (CFA) was conducted in R using the lavaan package. A robust maximum likelihood estimator (MLR) with respondent-level cluster correction was used to account for repeated ratings from the same respondent. The three-factor model showed good fit (CFI = 0.973, TLI = 0.965, RMSEA = 0.067, SRMR = 0.026). Standardized factor loadings, composite reliability (CR), average variance extracted (AVE), and the inter-factor correlation matrix are reported in Appendix B1–B3. All standardized loadings ranged from 0.770 to 0.864, indicating satisfactory explanatory power of the observed items for their corresponding latent constructs. The CR values for formal representation, usage inference, and meaning construction were 0.869, 0.877, and 0.901, respectively, and all AVE values exceeded 0.50. Although the correlations among dimensions indicate a certain degree of coupling, comparison with the two-factor and one-factor models (Table 10) shows that the three-factor model performs substantially better in both fit indices and information criteria. It was therefore retained for subsequent product-level scoring and user-side comparison.

**Table 10:** Fit comparison of the three-factor, two-factor, and one-factor CFA models.

	CFI	TLI	RMSEA	SRMR	AIC	BIC
Three-factor model	0.973	0.965	0.067	0.026	22142.96	22321.56
Two-factor model	0.951	0.939	0.089	0.035	22278.77	22448.20
One-factor model	0.904	0.882	0.123	0.050	22567.70	22732.56

The three-factor model comprises formal representation, usage inference, and meaning construction. The two-factor model combines usage inference and meaning construction into one factor, whereas the one-factor model loads all items onto a single factor. CFI = Comparative Fit Index; TLI = Tucker–Lewis Index; RMSEA = Root Mean Square Error of Approximation; SRMR = Standardized Root Mean Square Residual; AIC = Akaike Information Criterion; BIC = Bayesian Information Criterion. Higher values of CFI and TLI generally indicate better fit, whereas lower values of RMSEA, SRMR, AIC, and BIC indicate better model performance.

To estimate product-level perceived cultural value score under the BIBD-based repeated-measures design, a linear mixed-effects model (LMM) was used to calculate estimated marginal means (EMMs) and 95% confidence intervals, from which the user-side ranking was derived. Let *y*_*ibp*_ denote the overall perceived cultural value score assigned by respondent *i* to product *p* in questionnaire package *b*, calculated as the mean of the 12 items, with higher values indicating better perceived cultural value. The model is given in Eq. (27). Table 11 reports the fitted EMMs, 95% confidence intervals, and the resulting user-side ranking. Compared with the expert-side DEMATEL–CRITIC–MULTIMOORA ranking, the two approaches were fully consistent in identifying the top three products and showed relatively high rank agreement (Spearman’s *ρ* = 0.829; Kendall’s *τ* = 0.733). These results suggest that the proposed method can provide preliminary support for identifying higher-performing perceived cultural value schemes across expert-side and user-side evidence. In other words, the ranking derived from the three-domain framework and the DEMATEL–CRITIC–MULTIMOORA procedure reflects structured expert judgment and is broadly consistent with user-side questionnaire evaluations under e-commerce visual-textual conditions. This provides external reference support for the interpretability and practical applicability of the method in concept screening and competitor comparison.

27 $$\:{y}_{ibp}={\beta\:}_{0}+{\beta\:}_{p}+{\mu\:}_{i}+{v}_{b}+{\epsilon\:}_{ibp}$$

where *β*_*0*_ is the overall intercept, *β*_*p*_ is the fixed effect of product *p*, *µ*_*i*_ is the respondent-level random intercept capturing individual differences in overall scoring tendency, *ν*_*b*_ is the questionnaire-package random intercept capturing systematic differences across packages, and *ε*_*ibp*_ is the residual term. Here, $\:{\mu\:}_{i}\sim\:N\left(0,{\sigma\:}_{\mu\:}^{2}\right)$,$\:\:{v}_{b}\sim\:N\left(0,{\sigma\:}_{v}^{2}\right)$, and $\:{\epsilon\:}_{ibp}\sim\:N\left(0,{\sigma\:}^{2}\right)$.

**Table 11:** Estimated marginal means, 95% confidence intervals, and user-side ranking of the assessed products.

	EMM	SE	95% LCL	95% UCL	Ranking
*P*_1_	5.280	0.097	5.091	5.470	6
*P*_2_	5.767	0.097	5.577	5.956	2
*P*_3_	5.860	0.097	5.671	6.049	1
*P*_4_	5.586	0.097	5.397	5.775	3
*P*_5_	5.539	0.097	5.349	5.728	4
*P*_6_	5.529	0.097	5.339	5.718	5

EMM = estimated marginal mean; SE = standard error; LCL = lower confidence limit; UCL = upper confidence limit. The confidence limits are based on the 95% confidence interval. Higher EMM values indicate better overall perceived cultural value performance.

The comparison with user-side questionnaire data provides external reference support for interpreting the model outputs. However, the Kendall’s *W* results indicate that expert aggregation should not be interpreted as a highly convergent consensus. The indicator-weight rankings reflected heterogeneous expert judgments, while the alternative rankings showed only moderate and statistically non-significant coordination. Therefore, the aggregated results are better understood as expert-weighted group judgments under heterogeneous opinions. This viewpoint is consistent with the core philosophy of MCDA. MCDA models sort out assessment logic and keep complete analytical records. They make preference trade-offs visible when different views emerge, rather than forcing consensus or seeking a single conclusion. Rather than delivering absolute objectivity, the model improves procedural transparency through standard workflows, explicit indicator rules, rigorous weighting procedures, and traceable ranking calculations. Uniform index rules, rigorous weighting methods and standard ranking calculations greatly boost process transparency. However, the model does not intend to define a fixed, universal criterion for cultural aesthetics. Therefore, the utility scores and rankings from this work are merely practical references. They support the assessment of perceived cultural value using online visual and textual content. Multiple factors including expert members, product samples, visual-textual materials and assessment settings will influence the final outcomes. These results cannot directly measure a product’s historical authenticity, material quality or inherent cultural value.

Our study also found minor rank changes among products that placed lower in the overall list. This difference stems from the inherent gaps between the two assessment mechanisms. It does not mean the model has flaws. Expert-led assessment pays close attention to correlations between indicators and weight distribution. It also easily picks out weak points of individual products. In contrast, ordinary users make judgments with limited information on e-commerce pages. They tend to rely on obvious visual clues and overall impressions. Such divergences are neither random noise nor assessment errors. Instead, they reveal differences among decision-makers. These differences may lie in academic backgrounds, ways of interpreting culture, and the ability to understand diverse and unstandardized forms of cultural translation. Overall, the results suggest that perceived cultural value in e-commerce contexts depends less on the simple accumulation of cultural elements than on the consistency among formal expression, usage cues, and meaning communication. From a socio-technical and value co-creation perspective, this finding further suggests that e-commerce visual-textual presentation functions as a technical interface through which cultural value propositions are organized and interpreted by users. The proposed method therefore has value not only for product ranking, but also for design diagnosis and presentation optimization.

## Conclusions

This study addresses the problem of how the cultural semantics embodied in CCPs can be organized into observable and comparable evidence for perceived cultural value assessment under e-commerce visual-textual presentation. To this end, a three-domain indicator framework, comprising formal representation, usage inference, and meaning construction, was developed and operationalized through observable indicators and evidence domains. A DEMATEL–CRITIC–MULTIMOORA hybrid model was then constructed to integrate inter-indicator influence information, alternative-performance information, and multi-perspective ranking results. The results indicate that the proposed indicator system shows reasonable structural coherence within the present case context, and that the model-based rankings are broadly consistent with the user-side perceived cultural value ranking derived from questionnaire-based assessment results under the same visual-textual materials. These findings suggest that the framework can support the structured differentiation of presentation-based perceived cultural value among CCPs under e-commerce visual-textual conditions. Overall, this study contributes a design-oriented perceived cultural value assessment framework for platform-based consumption contexts and an integrated analytical path linking indicator construction, hybrid assessment, and user-side comparison, thereby providing a structured and comparable decision-support reference for online competitor analysis, page optimization, and design decision-making.

Several limitations should be acknowledged. First, this study used standardized static visual-textual materials to improve comparability, whereas authentic e-commerce pages contain more complex contextual cues, such as lifestyle images, promotional rhetoric, user-generated content, price signals, brand familiarity, seller reputation, and platform recommendation mechanisms. Visual materials also cannot fully replace tactile experience or actual product performance. Therefore, the framework still requires further testing in more heterogeneous and less controlled platform environments. Second, although evidence domains and concise explanations were provided to support non-expert interpretation, this study did not conduct an independent pilot test or cognitive interview to verify whether lay respondents understood all indicators consistently. The user-side comparison was also based on questionnaire data rather than behavioral indicators such as clicks, dwell time, conversion, or actual sales. Thus, the current external reference evidence mainly supports consistency with user-side perceived cultural value assessments rather than actual market behavior. Third, five product-evaluation experts were selected from the eight experts involved in indicator validation, which may introduce confirmation bias. Moreover, Kendall’s *W* suggests that expert aggregation should be interpreted as expert-weighted group assessment under heterogeneous preferences rather than highly convergent consensus or objective cultural quality judgment. Finally, the case was limited to six aromatherapy burners, restricting generalizability to other CCP categories. Cultural taste and “contemporary translation” are also time-sensitive, so the results should be viewed as a context-specific decision-support assessment rather than a timeless perceived cultural value judgment.

Future research may proceed in four directions. First, the assessment context can be extended from controlled static materials to authentic e-commerce pages by incorporating broader platform-related contextual cues into the assessment process. Second, external reference support can be strengthened by combining expert judgment, user perception, pilot tests or cognitive interviews, and behavioral evidence related to browsing, engagement, and conversion. Third, future studies should introduce independent external expert panels and examine whether expert disagreement reflects not only limited consensus but also divergent professional priorities, or innovative and controversial forms of cultural translation. Fourth, the framework should be tested across broader product categories, cultural themes, user groups, and platform types. Cross-cultural and longitudinal studies may further examine how perceived cultural value judgments vary across cultural value orientations, generations, and platform trends, and whether indicators such as “contemporary translation” require periodic updating. In the longer term, if multi-period and behavior-linked datasets become available, probabilistic models or agent-based simulations may be used to explore how perceived cultural value judgments and consensus patterns evolve across users, platforms, and cultural trends.

## Supplementary Information

Below is the link to the electronic supplementary material.

Supplementary Material 1

## Data Availability

Data are available on request to corresponding author.

## Appendix A

See Tables A1, A2, B1, B2, B3.

**Table A1:** The first-round revision records of content validity testing.

Primary indicator	Secondary indicator	$\:{\kappa\:}_{\mathrm{R}\mathrm{e}\mathrm{l}}^{\ast\:}$	$\:{\kappa\:}_{\mathrm{C}\mathrm{l}\mathrm{a}}^{\ast\:}$	$\:{\kappa\:}_{\mathrm{R}\mathrm{e}\mathrm{p}}^{\ast\:}$	$\:{\kappa\:}_{\mathrm{O}\mathrm{p}\mathrm{e}}^{\ast\:}$	Retain/Revise/Delete	Suggestion
Formal representation (*C*^1^)	Morphological composition ($\:{C}_{1}^{1}$)	1.000	0.719	0.719	0.719	Revise	Definition unclear.
	Color scheme ($\:{C}_{2}^{1}$)	1.000	0.719	0.719	0.871	Revise	Name to be adjusted; scheme may be revised to organization; definition unclear.
	Material expression ($\:{C}_{3}^{1}$)	0.871	0.719	0.719	0.871	Revise	Definition unclear.
	Graphic symbols ($\:{C}_{4}^{1}$)	1.000	0.871	0.719	1.000	Revise	Name to be adjusted; the term symbols is repetitive, and the name should follow a noun-based pattern; definition unclear.
	Craft details ($\:{C}_{5}^{1}$)	0.520	0.520	0.312	0.520	Delete	Overlaps with Material expression ($\:{C}_{3}^{1}$); suggested for deletion.
Usage inference (*C*^2^)	Functional logic ($\:{C}_{1}^{2}$)	1.000	0.719	0.719	0.871	Revise	Name to be adjusted; logic may be revised to coherence; definition unclear.
	Context fit ($\:{C}_{2}^{2}$)	1.000	0.719	0.719	0.719	Revise	Definition unclear.
	Interaction path ($\:{C}_{3}^{2}$)	1.000	0.871	0.719	0.871	Revise	Name to be adjusted; interaction path may be revised to use guidance.
	Cultural carrier fit ($\:{C}_{4}^{2}$)	1.000	0.520	0.719	0.719	Revise	Definition unclear.
	Experience expectation ($\:{C}_{5}^{2}$)	0.719	0.871	0.312	0.312	Delete	Too synthetic and overlapping with other indicators; suggested for deletion.
Meaning construction (*C*^3^)	Cultural symbols ($\:{C}_{1}^{3}$)	0.719	1.000	0.520	1.000	Delete	Overlaps with Graphic symbols ($\:{C}_{4}^{1}$); suggested for deletion.
	Value narrative ($\:{C}_{2}^{3}$)	1.000	0.520	0.719	0.520	Revise	Definition unclear.
	Emotional resonance ($\:{C}_{3}^{3}$)	1.000	0.520	0.719	0.719	Revise	Definition unclear.
	Identity recognition ($\:{C}_{4}^{3}$)	1.000	0.520	0.719	0.520	Revise	Definition unclear.
	Contemporary translation ($\:{C}_{5}^{3}$)	1.000	0.719	0.719	0.719	Revise	Definition unclear.

**Table A2:** The second-round revision records of content validity testing.

Primary indicator	Secondary indicator	Indicator definition	Evidence domain	$\:{\kappa\:}_{\mathrm{R}\mathrm{e}\mathrm{l}}^{\ast\:}$	$\:{\kappa\:}_{\mathrm{C}\mathrm{l}\mathrm{a}}^{\ast\:}$	$\:{\kappa\:}_{\mathrm{R}\mathrm{e}\mathrm{p}}^{\ast\:}$	$\:{\kappa\:}_{\mathrm{O}\mathrm{p}\mathrm{e}}^{\ast\:}$	Retain/Revise/Delete	Suggestion
Formal representation (*C*^1^)	Morphological composition ($\:{C}_{1}^{1}$)	Stable form structure supporting recognition of cultural prototypes.	Overall proportion; contour framework; compositional order	1.000	1.000	0.871	1.000	Retain	
	Color organization ($\:{C}_{2}^{1}$)	Coordinated color hierarchy supporting primary–secondary differentiation and stylistic consistency.	Primary–secondary color scheme; area proportion; color contrast	1.000	0.871	1.000	0.871	Retain	Replace “area proportion” with “color-block proportion”.
	Material expression ($\:{C}_{3}^{1}$)	Material and process cues supporting texture expectation and cultural character.	Material and process cues; texture cues; gloss cues	0.871	0.871	1.000	1.000	Retain	
	Graphic composition ($\:{C}_{4}^{1}$)	Ordered graphic elements fitting layout rules and overall style.	Graphic motifs; layout mode; overall style	1.000	1.000	1.000	0.871	Retain	Replace “overall style” with “organizational rules”.
Usage inference (*C*^2^)	Functional coherence ($\:{C}_{1}^{2}$)	Coherent functional structure linking key components and use flow.	Functional zones; key components; connection modes	1.000	1.000	1.000	1.000	Retain	
	Use guidance ($\:{C}_{2}^{2}$)	Controllable use process with explicit feedback and basic fault tolerance.	Entry controls; operational feedback; tolerance cues	1.000	0.871	0.871	0.871	Retain	
	Environmental fit ($\:{C}_{3}^{2}$)	Feasible fit with usage context, maintenance, and safety requirements.	Placement form; maintainability; safety boundaries	1.000	1.000	0.871	0.871	Retain	Replace “maintainability” with “maintenance mode”.
	Cultural carrier fit ($\:{C}_{4}^{2}$)	Appropriate fit between cultural expression and product carrier.	Cultural cues; product function; fit rationale	1.000	0.871	0.871	0.719	Retain	Replace “product function” with “carrier use”, and “fit rationale” with “mapping expressions”.
Meaning construction (*C*^3^)	Value narrative ($\:{C}_{1}^{3}$)	Coherent value-oriented narrative structure with internal consistency.	Symbolic cues; value claims; touchpoint consistency	1.000	0.871	1.000	0.871	Retain	Replace “narrative structure” with “symbolic cues” for greater clarity.
	Emotional resonance ($\:{C}_{2}^{3}$)	Emotional cues supporting atmosphere formation and resonance.	Emotional cues; atmosphere building; emotional articulation	0.871	1.000	1.000	1.000	Retain	
	Identity recognition ($\:{C}_{3}^{3}$)	Identity cues reinforcing value stance and contextual expression.	Identity markers; value stance; contextual expression	1.000	1.000	1.000	0.871	Retain	Replace “value stance” with “stance expression”.
	Contemporary translation ($\:{C}_{4}^{3}$)	Appropriate translation strategy preserving core semantics while fitting contemporary aesthetics.	Translation strategy; semantic retention; contemporary vocabulary	1.000	1.000	1.000	1.000	Retain	

## Appendix B

**Table B1:** Standardized factor loadings of the three-factor measurement model.

Indicator	Standardized Loading	Indicator	Standardized Loading	Indicator	Standardized Loading	Indicator	Standardized Loading
$\:{C}_{1}^{1}$	0.773	$\:{C}_{2}^{1}$	0.772	$\:{C}_{3}^{1}$	0.783	$\:{C}_{4}^{1}$	0.830
$\:{C}_{1}^{2}$	0.827	$\:{C}_{2}^{2}$	0.770	$\:{C}_{3}^{2}$	0.796	$\:{C}_{4}^{2}$	0.807
$\:{C}_{1}^{3}$	0.782	$\:{C}_{2}^{3}$	0.864	$\:{C}_{3}^{3}$	0.861	$\:{C}_{4}^{3}$	0.824

**Table B2:** Composite reliability and average variance extracted of the three dimensions.

Primary indicator	CR	AVE
*C* ^1^	0.869	0.624
*C* ^2^	0.877	0.640
*C* ^3^	0.901	0.695

**Table B3:** Inter-dimensional correlation matrix of the three dimensions.

Primary indicator	*C* ^1^	*C* ^2^	*C* ^3^
*C* ^1^	0.790	0.872	0.772
*C* ^2^	0.872	0.800	0.909
*C* ^3^	0.772	0.909	0.833

## Code availability

No custom code or bespoke computational tool was developed for this study. The calculations were performed using Microsoft Excel and SPSSAU according to the equations described in the Methods section. The intermediate matrices and procedural calculation materials used to support the reproducibility of the analysis are provided in the Supplementary Information, while the main calculation results are reported in the manuscript.
