# Supplementary material for: A hybrid method for assessing the perceived cultural value of cultural and creative products in e-commerce visual-textual presentation
Source: Sci Rep. 2026 Jun 23;16:28671. doi: 10.1038/s41598-026-58779-2 (PMC13575253; doi:10.1038/s41598-026-58779-2)
Supplement: Supplementary file 1 [file 41598_2026_58779_MOESM1_ESM.docx]

**Table S1. Individual direct-relation matrices provided by five experts.**

|  |  | $C_{1}^{1}$ | $C_{2}^{1}$ | $C_{3}^{1}$ | $C_{4}^{1}$ | $C_{1}^{2}$ | $C_{2}^{2}$ | $C_{3}^{2}$ | $C_{4}^{2}$ | $C_{1}^{3}$ | $C_{2}^{3}$ | $C_{3}^{3}$ | $C_{4}^{3}$ |
| --- | --- | --- | --- | --- | --- | --- | --- | --- | --- | --- | --- | --- | --- |
| *E*_1_ | $C_{1}^{1}$ | 0 | 5 | 7 | 7 | 6 | 7 | 7 | 7 | 7 | 7 | 5 | 7 |
|  | $C_{2}^{1}$ | 7 | 0 | 5 | 5 | 6 | 6 | 7 | 7 | 7 | 7 | 4 | 5 |
|  | $C_{3}^{1}$ | 6 | 6 | 0 | 5 | 4 | 6 | 7 | 7 | 7 | 7 | 6 | 7 |
|  | $C_{4}^{1}$ | 7 | 5 | 5 | 0 | 7 | 7 | 7 | 7 | 7 | 7 | 7 | 6 |
|  | $C_{1}^{2}$ | 6 | 6 | 5 | 7 | 0 | 5 | 6 | 7 | 7 | 4 | 5 | 5 |
|  | $C_{2}^{2}$ | 6 | 6 | 7 | 5 | 6 | 0 | 4 | 5 | 5 | 4 | 5 | 6 |
|  | $C_{3}^{2}$ | 7 | 7 | 7 | 7 | 5 | 5 | 0 | 6 | 7 | 7 | 5 | 7 |
|  | $C_{4}^{2}$ | 7 | 7 | 7 | 7 | 6 | 5 | 7 | 0 | 7 | 7 | 6 | 7 |
|  | $C_{1}^{3}$ | 7 | 7 | 6 | 7 | 5 | 4 | 6 | 7 | 0 | 5 | 5 | 6 |
|  | $C_{2}^{3}$ | 7 | 6 | 7 | 7 | 5 | 6 | 6 | 7 | 6 | 0 | 6 | 7 |
|  | $C_{3}^{3}$ | 6 | 6 | 6 | 7 | 5 | 6 | 5 | 7 | 6 | 7 | 0 | 7 |
|  | $C_{4}^{3}$ | 7 | 7 | 7 | 7 | 5 | 6 | 7 | 7 | 7 | 7 | 4 | 0 |
|  |  | $C_{1}^{1}$ | $C_{2}^{1}$ | $C_{3}^{1}$ | $C_{4}^{1}$ | $C_{1}^{2}$ | $C_{2}^{2}$ | $C_{3}^{2}$ | $C_{4}^{2}$ | $C_{1}^{3}$ | $C_{2}^{3}$ | $C_{3}^{3}$ | $C_{4}^{3}$ |
| *E*_2_ | $C_{1}^{1}$ | 0 | 7 | 6 | 6 | 5 | 6 | 7 | 5 | 5 | 5 | 5 | 4 |
|  | $C_{2}^{1}$ | 7 | 0 | 6 | 5 | 5 | 6 | 7 | 5 | 4 | 6 | 6 | 3 |
|  | $C_{3}^{1}$ | 7 | 5 | 0 | 5 | 5 | 7 | 7 | 4 | 4 | 6 | 6 | 3 |
|  | $C_{4}^{1}$ | 7 | 6 | 5 | 0 | 6 | 5 | 7 | 4 | 5 | 5 | 5 | 4 |
|  | $C_{1}^{2}$ | 6 | 5 | 6 | 6 | 0 | 6 | 6 | 5 | 4 | 6 | 4 | 4 |
|  | $C_{2}^{2}$ | 6 | 7 | 7 | 5 | 6 | 0 | 7 | 3 | 5 | 5 | 4 | 4 |
|  | $C_{3}^{2}$ | 5 | 7 | 7 | 5 | 6 | 6 | 0 | 4 | 5 | 5 | 5 | 4 |
|  | $C_{4}^{2}$ | 5 | 7 | 6 | 4 | 6 | 6 | 6 | 0 | 5 | 5 | 4 | 4 |
|  | $C_{1}^{3}$ | 5 | 6 | 6 | 4 | 5 | 7 | 6 | 3 | 0 | 5 | 4 | 4 |
|  | $C_{2}^{3}$ | 5 | 7 | 5 | 4 | 5 | 5 | 5 | 3 | 5 | 0 | 3 | 4 |
|  | $C_{3}^{3}$ | 4 | 7 | 5 | 4 | 5 | 5 | 5 | 4 | 5 | 5 | 0 | 4 |
|  | $C_{4}^{3}$ | 4 | 6 | 5 | 4 | 4 | 4 | 4 | 4 | 6 | 6 | 5 | 0 |
|  |  | $C_{1}^{1}$ | $C_{2}^{1}$ | $C_{3}^{1}$ | $C_{4}^{1}$ | $C_{1}^{2}$ | $C_{2}^{2}$ | $C_{3}^{2}$ | $C_{4}^{2}$ | $C_{1}^{3}$ | $C_{2}^{3}$ | $C_{3}^{3}$ | $C_{4}^{3}$ |
| *E*_3_ | $C_{1}^{1}$ | 0 | 5 | 5 | 5 | 5 | 3 | 5 | 5 | 5 | 4 | 4 | 4 |
|  | $C_{2}^{1}$ | 4 | 0 | 5 | 5 | 3 | 2 | 4 | 4 | 5 | 7 | 5 | 5 |
|  | $C_{3}^{1}$ | 4 | 5 | 0 | 3 | 5 | 3 | 4 | 5 | 5 | 6 | 5 | 5 |
|  | $C_{4}^{1}$ | 5 | 6 | 4 | 0 | 3 | 2 | 3 | 5 | 5 | 5 | 5 | 5 |
|  | $C_{1}^{2}$ | 4 | 2 | 3 | 2 | 0 | 7 | 6 | 3 | 3 | 3 | 2 | 3 |
|  | $C_{2}^{2}$ | 2 | 2 | 2 | 2 | 6 | 0 | 5 | 2 | 2 | 4 | 2 | 2 |
|  | $C_{3}^{2}$ | 3 | 2 | 3 | 2 | 5 | 4 | 0 | 3 | 2 | 2 | 2 | 3 |
|  | $C_{4}^{2}$ | 5 | 5 | 5 | 5 | 4 | 3 | 4 | 0 | 7 | 6 | 5 | 6 |
|  | $C_{1}^{3}$ | 3 | 4 | 3 | 5 | 3 | 3 | 3 | 6 | 0 | 6 | 7 | 5 |
|  | $C_{2}^{3}$ | 2 | 5 | 3 | 4 | 2 | 4 | 2 | 5 | 5 | 0 | 6 | 4 |
|  | $C_{3}^{3}$ | 2 | 4 | 3 | 3 | 2 | 2 | 2 | 5 | 6 | 6 | 0 | 4 |
|  | $C_{4}^{3}$ | 5 | 5 | 5 | 5 | 4 | 3 | 4 | 6 | 7 | 6 | 6 | 0 |
|  |  | $C_{1}^{1}$ | $C_{2}^{1}$ | $C_{3}^{1}$ | $C_{4}^{1}$ | $C_{1}^{2}$ | $C_{2}^{2}$ | $C_{3}^{2}$ | $C_{4}^{2}$ | $C_{1}^{3}$ | $C_{2}^{3}$ | $C_{3}^{3}$ | $C_{4}^{3}$ |
| *E*_4_ | $C_{1}^{1}$ | 0 | 5 | 2 | 3 | 5 | 7 | 2 | 2 | 5 | 3 | 3 | 1 |
|  | $C_{2}^{1}$ | 2 | 0 | 4 | 5 | 4 | 3 | 3 | 5 | 4 | 5 | 4 | 4 |
|  | $C_{3}^{1}$ | 3 | 1 | 0 | 4 | 3 | 6 | 4 | 6 | 3 | 2 | 2 | 5 |
|  | $C_{4}^{1}$ | 4 | 2 | 5 | 0 | 2 | 5 | 2 | 1 | 2 | 1 | 4 | 2 |
|  | $C_{1}^{2}$ | 3 | 5 | 4 | 1 | 0 | 2 | 4 | 2 | 5 | 2 | 3 | 3 |
|  | $C_{2}^{2}$ | 3 | 3 | 3 | 2 | 4 | 0 | 3 | 3 | 6 | 4 | 5 | 5 |
|  | $C_{3}^{2}$ | 2 | 4 | 2 | 4 | 2 | 1 | 0 | 5 | 3 | 2 | 2 | 3 |
|  | $C_{4}^{2}$ | 4 | 3 | 3 | 3 | 1 | 4 | 2 | 0 | 2 | 3 | 1 | 5 |
|  | $C_{1}^{3}$ | 2 | 2 | 1 | 2 | 5 | 5 | 1 | 2 | 0 | 6 | 5 | 2 |
|  | $C_{2}^{3}$ | 1 | 4 | 3 | 4 | 6 | 2 | 4 | 3 | 3 | 0 | 6 | 3 |
|  | $C_{3}^{3}$ | 2 | 3 | 3 | 3 | 5 | 4 | 5 | 4 | 1 | 4 | 0 | 4 |
|  | $C_{4}^{3}$ | 3 | 2 | 4 | 2 | 4 | 3 | 4 | 5 | 5 | 5 | 4 | 0 |
|  |  | $C_{1}^{1}$ | $C_{2}^{1}$ | $C_{3}^{1}$ | $C_{4}^{1}$ | $C_{1}^{2}$ | $C_{2}^{2}$ | $C_{3}^{2}$ | $C_{4}^{2}$ | $C_{1}^{3}$ | $C_{2}^{3}$ | $C_{3}^{3}$ | $C_{4}^{3}$ |
| *E*_5_ | $C_{1}^{1}$ | 0 | 5 | 5 | 5 | 5 | 6 | 7 | 5 | 6 | 6 | 6 | 7 |
|  | $C_{2}^{1}$ | 6 | 0 | 4 | 6 | 3 | 5 | 6 | 5 | 5 | 5 | 5 | 6 |
|  | $C_{3}^{1}$ | 4 | 4 | 0 | 4 | 5 | 5 | 6 | 4 | 4 | 5 | 3 | 5 |
|  | $C_{4}^{1}$ | 5 | 4 | 4 | 0 | 3 | 5 | 6 | 5 | 5 | 5 | 6 | 5 |
|  | $C_{1}^{2}$ | 5 | 3 | 5 | 4 | 0 | 5 | 4 | 3 | 3 | 5 | 5 | 6 |
|  | $C_{2}^{2}$ | 6 | 3 | 5 | 3 | 5 | 0 | 3 | 3 | 3 | 3 | 3 | 5 |
|  | $C_{3}^{2}$ | 7 | 5 | 6 | 5 | 4 | 4 | 0 | 6 | 6 | 5 | 5 | 6 |
|  | $C_{4}^{2}$ | 6 | 5 | 4 | 6 | 3 | 3 | 5 | 0 | 5 | 5 | 5 | 5 |
|  | $C_{1}^{3}$ | 5 | 5 | 4 | 6 | 3 | 3 | 5 | 6 | 0 | 6 | 6 | 5 |
|  | $C_{2}^{3}$ | 6 | 6 | 5 | 6 | 5 | 4 | 5 | 6 | 6 | 0 | 6 | 6 |
|  | $C_{3}^{3}$ | 7 | 5 | 5 | 6 | 4 | 3 | 5 | 6 | 6 | 6 | 0 | 5 |
|  | $C_{4}^{3}$ | 7 | 5 | 5 | 5 | 6 | 5 | 6 | 7 | 5 | 5 | 5 | 0 |

**Table S2. The group direct relation matrix *Z*.**

|  | $C_{1}^{1}$ | $C_{2}^{1}$ | $C_{3}^{1}$ | $C_{4}^{1}$ | $C_{1}^{2}$ | $C_{2}^{2}$ | $C_{3}^{2}$ | $C_{4}^{2}$ | $C_{1}^{3}$ | $C_{2}^{3}$ | $C_{3}^{3}$ | $C_{4}^{3}$ |
| --- | --- | --- | --- | --- | --- | --- | --- | --- | --- | --- | --- | --- |
| $C_{1}^{1}$ | 0.000 | 5.400 | 4.960 | 5.140 | 5.150 | 5.580 | 5.600 | 4.760 | 5.520 | 4.910 | 4.610 | 4.570 |
| $C_{2}^{1}$ | 5.130 | 0.000 | 4.800 | 5.220 | 4.030 | 4.240 | 5.310 | 5.050 | 4.920 | 6.000 | 4.870 | 4.640 |
| $C_{3}^{1}$ | 4.720 | 4.210 | 0.000 | 4.100 | 4.490 | 5.230 | 5.490 | 5.060 | 4.520 | 5.210 | 4.370 | 4.900 |
| $C_{4}^{1}$ | 5.520 | 4.690 | 4.530 | 0.000 | 4.020 | 4.550 | 4.880 | 4.380 | 4.760 | 4.580 | 5.340 | 4.410 |
| $C_{1}^{2}$ | 4.740 | 3.960 | 4.520 | 3.810 | 0.000 | 5.160 | 5.200 | 3.820 | 4.160 | 4.010 | 3.690 | 4.160 |
| $C_{2}^{2}$ | 4.460 | 4.000 | 4.590 | 3.270 | 5.420 | 0.000 | 4.450 | 3.050 | 3.990 | 3.980 | 3.610 | 4.200 |
| $C_{3}^{2}$ | 4.700 | 4.770 | 4.880 | 4.370 | 4.440 | 4.010 | 0.000 | 4.670 | 4.410 | 4.010 | 3.710 | 4.460 |
| $C_{4}^{2}$ | 5.340 | 5.340 | 4.920 | 4.960 | 3.940 | 4.080 | 4.710 | 0.000 | 5.260 | 5.190 | 4.230 | 5.350 |
| $C_{1}^{3}$ | 4.260 | 4.710 | 3.910 | 4.780 | 4.060 | 4.310 | 4.130 | 4.830 | 0.000 | 5.650 | 5.520 | 4.410 |
| $C_{2}^{3}$ | 4.050 | 5.590 | 4.440 | 4.890 | 4.430 | 4.140 | 4.220 | 4.760 | 5.010 | 0.000 | 5.400 | 4.710 |
| $C_{3}^{3}$ | 4.100 | 4.940 | 4.290 | 4.460 | 4.030 | 3.780 | 4.250 | 5.140 | 4.900 | 5.590 | 0.000 | 4.670 |
| $C_{4}^{3}$ | 5.180 | 4.960 | 5.120 | 4.560 | 4.590 | 4.090 | 4.890 | 5.790 | 6.000 | 5.750 | 4.920 | 0.000 |

**Table S3. The normalized direct relation matrix *D*.**

|  | $C_{1}^{1}$ | $C_{2}^{1}$ | $C_{3}^{1}$ | $C_{4}^{1}$ | $C_{1}^{2}$ | $C_{2}^{2}$ | $C_{3}^{2}$ | $C_{4}^{2}$ | $C_{1}^{3}$ | $C_{2}^{3}$ | $C_{3}^{3}$ | $C_{4}^{3}$ |
| --- | --- | --- | --- | --- | --- | --- | --- | --- | --- | --- | --- | --- |
| $C_{1}^{1}$ | 0.000 | 0.096 | 0.088 | 0.092 | 0.092 | 0.099 | 0.100 | 0.085 | 0.098 | 0.087 | 0.082 | 0.081 |
| $C_{2}^{1}$ | 0.091 | 0.000 | 0.085 | 0.093 | 0.072 | 0.075 | 0.095 | 0.090 | 0.088 | 0.107 | 0.087 | 0.083 |
| $C_{3}^{1}$ | 0.084 | 0.075 | 0.000 | 0.073 | 0.080 | 0.093 | 0.098 | 0.090 | 0.080 | 0.093 | 0.078 | 0.087 |
| $C_{4}^{1}$ | 0.098 | 0.084 | 0.081 | 0.000 | 0.072 | 0.081 | 0.087 | 0.078 | 0.085 | 0.082 | 0.095 | 0.079 |
| $C_{1}^{2}$ | 0.084 | 0.071 | 0.080 | 0.068 | 0.000 | 0.092 | 0.093 | 0.068 | 0.074 | 0.071 | 0.066 | 0.074 |
| $C_{2}^{2}$ | 0.079 | 0.071 | 0.082 | 0.058 | 0.096 | 0.000 | 0.079 | 0.054 | 0.071 | 0.071 | 0.064 | 0.075 |
| $C_{3}^{2}$ | 0.084 | 0.085 | 0.087 | 0.078 | 0.079 | 0.071 | 0.000 | 0.083 | 0.079 | 0.071 | 0.066 | 0.079 |
| $C_{4}^{2}$ | 0.095 | 0.095 | 0.088 | 0.088 | 0.070 | 0.073 | 0.084 | 0.000 | 0.094 | 0.092 | 0.075 | 0.095 |
| $C_{1}^{3}$ | 0.076 | 0.084 | 0.070 | 0.085 | 0.072 | 0.077 | 0.074 | 0.086 | 0.000 | 0.101 | 0.098 | 0.079 |
| $C_{2}^{3}$ | 0.072 | 0.100 | 0.079 | 0.087 | 0.079 | 0.074 | 0.075 | 0.085 | 0.089 | 0.000 | 0.096 | 0.084 |
| $C_{3}^{3}$ | 0.073 | 0.088 | 0.076 | 0.079 | 0.072 | 0.067 | 0.076 | 0.092 | 0.087 | 0.100 | 0.000 | 0.083 |
| $C_{4}^{3}$ | 0.092 | 0.088 | 0.091 | 0.081 | 0.082 | 0.073 | 0.087 | 0.103 | 0.107 | 0.102 | 0.088 | 0.000 |

**Table S4. The total relation matrix *T*.**

|  | $C_{1}^{1}$ | $C_{2}^{1}$ | $C_{3}^{1}$ | $C_{4}^{1}$ | $C_{1}^{2}$ | $C_{2}^{2}$ | $C_{3}^{2}$ | $C_{4}^{2}$ | $C_{1}^{3}$ | $C_{2}^{3}$ | $C_{3}^{3}$ | $C_{4}^{3}$ |
| --- | --- | --- | --- | --- | --- | --- | --- | --- | --- | --- | --- | --- |
| $C_{1}^{1}$ | 0.905 | 1.002 | 0.967 | 0.950 | 0.932 | 0.947 | 1.011 | 0.971 | 1.016 | 1.030 | 0.953 | 0.954 |
| $C_{2}^{1}$ | 0.963 | 0.889 | 0.939 | 0.927 | 0.890 | 0.902 | 0.980 | 0.951 | 0.981 | 1.020 | 0.932 | 0.930 |
| $C_{3}^{1}$ | 0.925 | 0.926 | 0.830 | 0.879 | 0.868 | 0.888 | 0.951 | 0.920 | 0.943 | 0.975 | 0.893 | 0.904 |
| $C_{4}^{1}$ | 0.930 | 0.926 | 0.897 | 0.804 | 0.854 | 0.870 | 0.934 | 0.902 | 0.939 | 0.958 | 0.901 | 0.889 |
| $C_{1}^{2}$ | 0.848 | 0.844 | 0.829 | 0.800 | 0.722 | 0.814 | 0.868 | 0.824 | 0.858 | 0.875 | 0.808 | 0.817 |
| $C_{2}^{2}$ | 0.811 | 0.810 | 0.797 | 0.760 | 0.779 | 0.698 | 0.823 | 0.779 | 0.821 | 0.840 | 0.774 | 0.785 |
| $C_{3}^{2}$ | 0.870 | 0.878 | 0.856 | 0.830 | 0.815 | 0.817 | 0.806 | 0.859 | 0.884 | 0.899 | 0.830 | 0.843 |
| $C_{4}^{2}$ | 0.955 | 0.964 | 0.930 | 0.912 | 0.878 | 0.889 | 0.960 | 0.858 | 0.975 | 0.997 | 0.912 | 0.930 |
| $C_{1}^{3}$ | 0.894 | 0.910 | 0.871 | 0.867 | 0.838 | 0.850 | 0.906 | 0.893 | 0.844 | 0.957 | 0.888 | 0.873 |
| $C_{2}^{3}$ | 0.908 | 0.939 | 0.896 | 0.884 | 0.859 | 0.863 | 0.924 | 0.908 | 0.943 | 0.883 | 0.902 | 0.894 |
| $C_{3}^{3}$ | 0.887 | 0.908 | 0.872 | 0.857 | 0.833 | 0.838 | 0.903 | 0.893 | 0.919 | 0.951 | 0.794 | 0.872 |
| $C_{4}^{3}$ | 0.989 | 0.995 | 0.969 | 0.941 | 0.922 | 0.924 | 0.999 | 0.987 | 1.023 | 1.043 | 0.957 | 0.879 |

**Table S5. Individual alternative assessment matrices provided by five experts.**

|  |  | $C_{1}^{1}$ | $C_{2}^{1}$ | $C_{3}^{1}$ | $C_{4}^{1}$ | $C_{1}^{2}$ | $C_{2}^{2}$ | $C_{3}^{2}$ | $C_{4}^{2}$ | $C_{1}^{3}$ | $C_{2}^{3}$ | $C_{3}^{3}$ | $C_{4}^{3}$ |
| --- | --- | --- | --- | --- | --- | --- | --- | --- | --- | --- | --- | --- | --- |
| *E*_1_ | *P*_1_ | 6 | 5 | 7 | 7 | 6 | 4 | 7 | 7 | 6 | 4 | 4 | 6 |
|  | *P*_2_ | 7 | 6 | 6 | 7 | 7 | 5 | 7 | 7 | 6 | 5 | 4 | 7 |
|  | *P*_3_ | 7 | 7 | 7 | 7 | 6 | 7 | 7 | 7 | 7 | 6 | 5 | 7 |
|  | *P*_4_ | 7 | 6 | 7 | 7 | 6 | 7 | 7 | 7 | 7 | 5 | 4 | 7 |
|  | *P*_5_ | 7 | 6 | 7 | 7 | 6 | 7 | 7 | 7 | 7 | 5 | 4 | 7 |
|  | *P*_6_ | 6 | 6 | 7 | 7 | 7 | 6 | 7 | 7 | 6 | 5 | 4 | 7 |
|  |  | $C_{1}^{1}$ | $C_{2}^{1}$ | $C_{3}^{1}$ | $C_{4}^{1}$ | $C_{1}^{2}$ | $C_{2}^{2}$ | $C_{3}^{2}$ | $C_{4}^{2}$ | $C_{1}^{3}$ | $C_{2}^{3}$ | $C_{3}^{3}$ | $C_{4}^{3}$ |
| *E*_2_ | *P*_1_ | 7 | 7 | 6 | 7 | 7 | 7 | 6 | 7 | 7 | 7 | 6 | 6 |
|  | *P*_2_ | 7 | 7 | 7 | 7 | 7 | 7 | 6 | 7 | 6 | 7 | 7 | 6 |
|  | *P*_3_ | 7 | 6 | 7 | 7 | 7 | 7 | 7 | 7 | 7 | 7 | 7 | 6 |
|  | *P*_4_ | 6 | 7 | 7 | 6 | 7 | 7 | 7 | 6 | 7 | 7 | 7 | 7 |
|  | *P*_5_ | 6 | 7 | 6 | 7 | 7 | 7 | 7 | 6 | 7 | 7 | 7 | 7 |
|  | *P*_6_ | 7 | 7 | 7 | 6 | 7 | 7 | 6 | 6 | 7 | 7 | 7 | 7 |
|  |  | $C_{1}^{1}$ | $C_{2}^{1}$ | $C_{3}^{1}$ | $C_{4}^{1}$ | $C_{1}^{2}$ | $C_{2}^{2}$ | $C_{3}^{2}$ | $C_{4}^{2}$ | $C_{1}^{3}$ | $C_{2}^{3}$ | $C_{3}^{3}$ | $C_{4}^{3}$ |
| *E*_3_ | *P*_1_ | 6 | 5 | 6 | 4 | 6 | 6 | 6 | 7 | 6 | 5 | 5 | 6 |
|  | *P*_2_ | 6 | 6 | 6 | 4 | 6 | 6 | 6 | 6 | 6 | 5 | 6 | 6 |
|  | *P*_3_ | 7 | 6 | 6 | 6 | 6 | 5 | 5 | 6 | 6 | 5 | 5 | 5 |
|  | *P*_4_ | 6 | 4 | 5 | 4 | 5 | 6 | 6 | 6 | 5 | 5 | 5 | 5 |
|  | *P*_5_ | 6 | 6 | 7 | 6 | 6 | 5 | 5 | 6 | 6 | 5 | 4 | 4 |
|  | *P*_6_ | 6 | 6 | 5 | 4 | 6 | 6 | 6 | 6 | 5 | 6 | 5 | 5 |
|  |  | $C_{1}^{1}$ | $C_{2}^{1}$ | $C_{3}^{1}$ | $C_{4}^{1}$ | $C_{1}^{2}$ | $C_{2}^{2}$ | $C_{3}^{2}$ | $C_{4}^{2}$ | $C_{1}^{3}$ | $C_{2}^{3}$ | $C_{3}^{3}$ | $C_{4}^{3}$ |
| *E*_4_ | *P*_1_ | 3 | 3 | 4 | 5 | 2 | 4 | 6 | 3 | 2 | 5 | 4 | 2 |
|  | *P*_2_ | 5 | 4 | 6 | 3 | 2 | 5 | 6 | 3 | 2 | 3 | 2 | 4 |
|  | *P*_3_ | 3 | 4 | 5 | 4 | 2 | 6 | 5 | 3 | 4 | 4 | 3 | 2 |
|  | *P*_4_ | 2 | 4 | 6 | 5 | 2 | 3 | 4 | 5 | 2 | 2 | 2 | 4 |
|  | *P*_5_ | 3 | 3 | 4 | 4 | 5 | 5 | 4 | 3 | 3 | 3 | 3 | 3 |
|  | *P*_6_ | 2 | 5 | 4 | 6 | 4 | 3 | 2 | 6 | 4 | 2 | 3 | 4 |
|  |  | $C_{1}^{1}$ | $C_{2}^{1}$ | $C_{3}^{1}$ | $C_{4}^{1}$ | $C_{1}^{2}$ | $C_{2}^{2}$ | $C_{3}^{2}$ | $C_{4}^{2}$ | $C_{1}^{3}$ | $C_{2}^{3}$ | $C_{3}^{3}$ | $C_{4}^{3}$ |
| *E*_5_ | *P*_1_ | 3 | 3 | 5 | 5 | 5 | 5 | 4 | 5 | 5 | 5 | 5 | 3 |
|  | *P*_2_ | 5 | 3 | 3 | 4 | 5 | 3 | 5 | 6 | 5 | 6 | 6 | 4 |
|  | *P*_3_ | 6 | 4 | 5 | 5 | 6 | 5 | 6 | 5 | 5 | 6 | 5 | 4 |
|  | *P*_4_ | 5 | 5 | 5 | 5 | 5 | 6 | 4 | 4 | 4 | 4 | 6 | 5 |
|  | *P*_5_ | 2 | 2 | 3 | 3 | 4 | 5 | 3 | 3 | 3 | 2 | 2 | 3 |
|  | *P*_6_ | 3 | 5 | 5 | 5 | 4 | 4 | 4 | 2 | 5 | 5 | 4 | 3 |

**Table S6. The group alternative assessment matrix *X*.**

|  | $C_{1}^{1}$ | $C_{2}^{1}$ | $C_{3}^{1}$ | $C_{4}^{1}$ | $C_{1}^{2}$ | $C_{2}^{2}$ | $C_{3}^{2}$ | $C_{4}^{2}$ | $C_{1}^{3}$ | $C_{2}^{3}$ | $C_{3}^{3}$ | $C_{4}^{3}$ |
| --- | --- | --- | --- | --- | --- | --- | --- | --- | --- | --- | --- | --- |
| *P*_1_ | 5.00 | 4.60 | 5.57 | 5.45 | 5.26 | 5.32 | 5.71 | 5.84 | 5.26 | 5.25 | 4.87 | 4.62 |
| *P*_2_ | 5.95 | 5.18 | 5.54 | 4.87 | 5.41 | 5.21 | 5.93 | 5.81 | 5.06 | 5.26 | 5.18 | 5.35 |
| *P*_3_ | 6.06 | 5.35 | 5.95 | 5.77 | 5.48 | 5.88 | 5.92 | 5.59 | 5.77 | 5.59 | 5.04 | 4.74 |
| *P*_4_ | 5.21 | 5.12 | 5.88 | 5.25 | 5.01 | 5.81 | 5.55 | 5.53 | 4.94 | 4.64 | 4.93 | 5.52 |
| *P*_5_ | 4.73 | 4.78 | 5.38 | 5.33 | 5.58 | 5.70 | 5.08 | 4.95 | 5.15 | 4.38 | 3.98 | 4.65 |
| *P*_6_ | 4.82 | 5.80 | 5.52 | 5.43 | 5.55 | 5.22 | 4.99 | 5.27 | 5.37 | 5.11 | 4.67 | 5.08 |

**Table S7. The standardized group alternative assessment matrix *X^*^*.**

|  | $C_{1}^{1}$ | $C_{2}^{1}$ | $C_{3}^{1}$ | $C_{4}^{1}$ | $C_{1}^{2}$ | $C_{2}^{2}$ | $C_{3}^{2}$ | $C_{4}^{2}$ | $C_{1}^{3}$ | $C_{2}^{3}$ | $C_{3}^{3}$ | $C_{4}^{3}$ |
| --- | --- | --- | --- | --- | --- | --- | --- | --- | --- | --- | --- | --- |
| *P*_1_ | 0.3836 | 0.3644 | 0.4029 | 0.4154 | 0.3988 | 0.3927 | 0.4206 | 0.4329 | 0.4079 | 0.4240 | 0.4147 | 0.3768 |
| *P*_2_ | 0.4565 | 0.4104 | 0.4007 | 0.3712 | 0.4101 | 0.3846 | 0.4368 | 0.4307 | 0.3923 | 0.4248 | 0.4411 | 0.4363 |
| *P*_3_ | 0.4650 | 0.4239 | 0.4304 | 0.4397 | 0.4154 | 0.4341 | 0.4360 | 0.4144 | 0.4474 | 0.4515 | 0.4292 | 0.3866 |
| *P*_4_ | 0.3997 | 0.4056 | 0.4253 | 0.4001 | 0.3798 | 0.4289 | 0.4088 | 0.4099 | 0.3830 | 0.3747 | 0.4198 | 0.4502 |
| *P*_5_ | 0.3629 | 0.3787 | 0.3892 | 0.4062 | 0.4230 | 0.4208 | 0.3742 | 0.3670 | 0.3993 | 0.3538 | 0.3389 | 0.3792 |
| *P*_6_ | 0.3698 | 0.4595 | 0.3993 | 0.4138 | 0.4207 | 0.3853 | 0.3675 | 0.3907 | 0.4164 | 0.4127 | 0.3977 | 0.4143 |

**Table S8. Original utility values generated by the three MULTIMOORA sub-models.**

|  | $\chi_{p}$ (RS) | $\delta_{p}$ (RP) | $\varphi_{p}$ (FMF) |
| --- | --- | --- | --- |
| *P*_1_ | 0.4014 | 0.0094 | 0.4009 |
| *P*_2_ | 0.4180 | 0.0054 | 0.4173 |
| *P*_3_ | 0.4305 | 0.0067 | 0.4300 |
| *P*_4_ | 0.4079 | 0.0066 | 0.4074 |
| *P*_5_ | 0.3814 | 0.0097 | 0.3807 |
| *P*_6_ | 0.4046 | 0.0090 | 0.4039 |
